# Estimating the population impact of new tuberculosis vaccines depending on efficacy against infectious asymptomatic tuberculosis: A modelling study

**DOI:** 10.1371/journal.pmed.1004595

**Published:** 2026-02-12

**Authors:** Hira Tanvir, Rebecca A. Clark, Tom Sumner, Katherine C. Horton, Tomos O. Prŷs-Jones, Roel Bakker, Kirankumar Rade, Vidya Mave, Mark Hatherill, Gavin J. Churchyard, Rein M. G. J. Houben, Richard G. White

**Affiliations:** 1 TB Modelling Group, TB Centre, and Centre for Mathematical Modelling of Infectious Diseases, Department of Infectious Disease Epidemiology, London School of Hygiene & Tropical Medicine, London, United Kingdom; 2 Vaccine Centre, London School of Hygiene & Tropical Medicine, London, United Kingdom; 3 KNCV Tuberculosis Foundation, The Hague, Netherlands; 4 Independent Consultant, New Delhi, India; 5 Byramjee–Jeejeebhoy Government Medical College, Johns Hopkins University Clinical Research Site, Pune, India; 6 South African Tuberculosis Vaccine Initiative, Institute of Infectious Disease and Molecular Medicine and Department of Pathology, University of Cape Town, Cape Town, South Africa; 7 Aurum Institute NPC, Johannesburg, South Africa; 8 School of Public Health, University of Witwatersrand, Johannesburg, South Africa; 9 Department of Medicine, Vanderbilt University, Nashville, Tennessee, United States of America; PLOS Medicine Editorial Board, UNITED STATES OF AMERICA

## Abstract

**Background:**

Tuberculosis (TB) remains a leading cause of infectious disease death. New TB vaccines are currently in late-stage trials and may be available before the end of the decade. Modelling predicts new TB vaccines may reduce global burden but rely on assumptions about vaccine efficacy by TB disease stage and TB natural history, which may be incorrect. We explored the sensitivity of estimates of the impact of new TB vaccines to uncertainties in efficacy by disease stage and natural history.

**Methods and findings:**

We developed a dynamic compartmental TB model for India, including early TB disease stages (non-infectious disease, infectious asymptomatic disease, and infectious symptomatic disease). Scenarios assumed 50% vaccine efficacy for 10 years and prevented progression to (a) only infectious symptomatic disease, or (b) any infectious disease (infectious asymptomatic disease and infectious symptomatic disease), or (c) any disease (non-infectious disease, infectious asymptomatic disease, and infectious symptomatic disease). We estimated impact on averting disease episodes over 2030–2050, compared to no-new-vaccine introduction. Results suggest, over 3 years, there was little difference in the proportion of cumulative symptomatic disease episodes averted by vaccines preventing only infectious symptomatic disease, any infectious disease, or any disease (1.6%, 2.3%, and 2.3%, respectively). However, over 20 years, compared to vaccines preventing only infectious symptomatic disease, vaccines preventing any infectious disease, or any disease, averted a markedly higher proportion of symptomatic disease episodes (7.3%, 19.4%, and 23.3%, respectively), due to preventing continued transmission from infectious asymptomatic disease. A key limitation with any mathematical modelling study is the uncertainty associated with the inputs, and further data collection is required to better understand the transmissibility, morbidity, and dynamics of asymptomatic disease, to improve modelling estimates and inform wider policy.

**Conclusions:**

Our modelling estimates that the population impact of new TB vaccines may depend on efficacy against infectious asymptomatic disease. TB vaccine trials should include analyses of participant sputum samples (collected during or at the end of trials and analysed at the end of trials) to enable better estimates of the potential value of new TB vaccines against infectious asymptomatic disease.

## Author summary

**Why was this study done?:**

- New tuberculosis (TB) vaccines may be available soon, with a recently completed phase 2b trial of the vaccine candidate M72/AS01_E_ demonstrating encouraging results of around 50% (95% confidence interval = 2.1%, 74.2%) impact on the incidence of symptomatic TB.
- Recent modelling studies suggest that introducing a new vaccine that prevents TB could yield substantial global health and economic benefits.
- With recognition of infectious asymptomatic TB, we aimed to explore the sensitivity of estimates of the impact of new TB vaccines to uncertainties in vaccine efficacy by TB disease stage.

**What did the researchers do and find?:**

- We used a mathematical model to explore three scenarios varying how the vaccine could prevent progression to disease: preventing progression to infectious symptomatic disease only, preventing progression to any infectious disease, or preventing progression to any disease.
- Results suggested that over the short term, all scenarios averted a similar proportion of cumulative symptomatic TB episodes.
- However, over the longer term, the differences became more pronounced, and vaccines preventing any infectious disease or any disease averted a higher proportion of symptomatic TB episodes compared to a vaccine that only prevented infectious symptomatic TB.

**What do these findings mean?:**

- This study suggests that the population-level impact of new TB vaccines may depend on the efficacy of the vaccine against asymptomatic TB.
- TB vaccine trials should measure impact against asymptomatic TB to enable better estimates of the potential full value of new TB vaccines.
- Study limitations include limited data availability to parameterise the natural history and infectivity of asymptomatic TB, which could be improved with further data collection.

## Abstract 3

Hira Tanvir and colleagues estimate the potential impact of new tuberculosis vaccines under three scenarios of efficacy and conclude that to achieve the greatest impact, vaccines must prevent asymptomatic and symptomatic disease.

## Introduction

Tuberculosis (TB) causes around 10.8 million new episodes and 1.25 million deaths annually [1]. The highest number of TB episodes and deaths occurs in India, with more than a quarter of global TB incidence and mortality [1]. There is evidence that infectious asymptomatic TB disease, defined as bacteriologically positive TB in people not reporting TB symptoms, contributes to transmission and may be a significant driver of the TB epidemic [2,3]. Data from national TB prevalence surveys suggests that infectious asymptomatic TB may account for at least 50% of prevalent bacteriologically positive disease [4]. Asymptomatic TB may also progress to infectious symptomatic disease and contribute to post TB morbidity [3–7]. Individuals with or without TB symptoms can also be bacteriologically negative or unconfirmed on culture tests, but present with macroscopic pathology indicative of disease [8,9]. It is possible that individuals may regress and progress through TB infection and disease stages throughout their lives [10].

New TB vaccines may be available soon, with a recently completed phase 2b trial of the vaccine candidate M72/AS01_E_ demonstrating encouraging results of around 50% (95% confidence interval = 2.1%, 74.2%) impact on the incidence of symptomatic disease [11]. The primary endpoint of this trial was efficacy against bacteriologically confirmed (infectious) symptomatic TB (previously referred to as *clinical TB*), but the impact on infectious asymptomatic TB was not measured [5,8,9]. The phase 3 trial for M72/AS01_E_ (NCT06062238) and the phase 2b trial for MTBVAC (NCT06272812) are ongoing, and similarly measuring the impact on infectious symptomatic TB as their primary endpoint, although may plan to measure impact on asymptomatic TB [12].

Recent modelling studies suggest that introducing a new vaccine that prevents TB could yield substantial global health and economic benefits [13–17]. However, these estimates did not incorporate current uncertainty in the natural history of asymptomatic TB or uncertainty in the efficacy of the vaccine by TB disease stage [13–17]. If new TB vaccines have no impact on progression to non-infectious and/or infectious asymptomatic TB, and/or infectious asymptomatic TB contributes to transmission differently than assumed, these estimates of the impact of new TB vaccines could be incorrect [5]. Incorporating uncertainty in the infectiousness of asymptomatic TB and the impact of the vaccine by disease stages is thus critical for understanding the potential impact of new TB vaccines. The expected impact of this uncertainty is unquantified. Our prior belief before modelling is that it will range between approximately no effect if asymptomatic TB is not infectious, to a marked effect if a large proportion of transmission is from individuals with asymptomatic TB.

We used a mathematical model to explore the sensitivity of estimates of the impact of new TB vaccines to uncertainties in vaccine efficacy by TB disease stage and infectiousness of asymptomatic TB.

## Methods

### Setting and empirical data

We used demographic projections for India by single age and year from the United Nations population data [18]. Estimates for TB incidence, TB case notifications, and TB mortality were from the World Health Organization (WHO) [19]. TB disease and infection prevalence estimates were from the India National TB Prevalence Survey and Pandey and colleagues, 2017 [20,21]. A full list of parameters and sources is in Table A in S1 Text.

### Model structure

**Fig 1:**
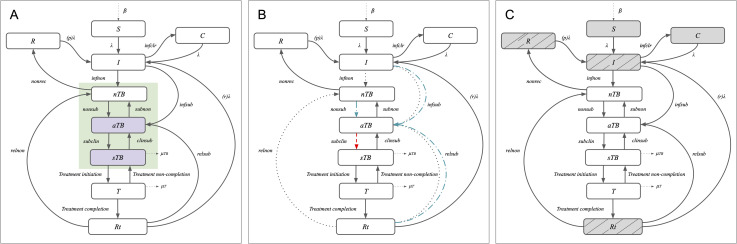
TB natural history model structure. **(A)** Illustration of core TB natural history model structure. TB disease states and parameters accounting for progression and regression between states are highlighted in green. TB disease states assumed to be infectious and contributing to transmission (infectious asymptomatic TB [aTB] and infectious symptomatic TB [sTB]) are highlighted in purple. **(B)** Scenarios for prevention of disease mechanism of action: preventing infectious symptomatic disease only (red dashed line), preventing any infectious disease (blue dash-dotted lines) or preventing any disease (grey dotted lines). **(C)** Illustration of host infectious status at time of vaccination required for efficacy—Default: any infection status (AI, all grey shaded boxes) or sensitivity analysis: current infection status only (CI, only grey shaded boxes with lines). Arrows represent the rate of flow per year from the origin compartment to the destination compartment, and natural history parameters (indicated along the arrows) are defined in Table A in S1 Text. *Abbreviations: S,  Susceptible; I, Infection; C, Cleared; R, Recovered; nTB, Non-infectious disease; aTB, Infectious asymptomatic disease; sTB, Infectious symptomatic Disease; T, On-treatment; Rt, Recovered after treatment.*

We used an age-stratified compartmental transmission model of TB, updating the natural history in line with recent modelling (Fig 1A) [13,22]. Our natural history structure consisted of nine compartments, modelling pathways from uninfected (S), to *Mycobacterium tuberculosis* (*Mtb)* infection (I, from which self-clearance of *Mtb* infection to a cleared compartment [C] was possible), through disease stages (described below), treatment (T), and recovery (R and Rt). See S1 Text for full details.

In line with emerging evidence and recent WHO guidance, we represented TB disease with three stages: infectious symptomatic TB (sTB), infectious asymptomatic TB (aTB), and non-infectious TB (nTB) [8,9]. We defined individuals with sTB as individuals with bacteriologically positive (infectious) TB reporting symptoms suggestive of TB during screening. We assumed additional TB mortality, regression to aTB, and treatment initiation from sTB. We defined aTB as bacteriologically positive (infectious) TB in individuals not reporting symptoms suggestive of TB during screening. We assumed no additional mortality from aTB, and we assumed the relative infectiousness of aTB compared to sTB was between 0.62 and 1 [2]. We assumed further disease progression from aTB to sTB, and regression from aTB to nTB. In our model, we defined nTB as bacteriologically negative (non-infectious) disease, which could be asymptomatic or symptomatic. We allowed for recovery from nTB, and further disease progression to aTB. We assumed no additional mortality from nTB.

### Statistical analysis

The model was calibrated using history matching with emulation [23] using the *hmer* R package [24] to obtain 500 parameter sets fitted to all 14 calibration targets (Table E in S1 Text).

### Calibration

Targets for all ages were the TB incidence rate in 2010 and 2020, mortality rate in 2010 and 2020, case notification rate in 2010 and 2020, infectious TB prevalence in 2015 and 2021, and infectious asymptomatic-to-symptomatic TB prevalence ratio in 2021 [4,18,20,21,25–28]. Targets for children were the incidence rate and case notification rate in 2020. Targets for adults were the TB incidence rate and case notification rate in 2020 and active TB prevalence rate in 2021. TB incidence rate targets were adjusted for double counting of episodes (representing multiple transitions from aTB to sTB within one year) during calibration by discounting the model output by 14% as in Horton and colleagues (2025) [29]. We assumed a uniform distribution between the low and high bounds for all calibration targets.

### No-new-vaccine scenario

We simulated a *no-new-vaccine* scenario from 1900 to 2050 assuming the constraints above and that the quality and coverage of non-vaccine TB interventions post 2022 would be maintained at 2022 levels, and no new TB vaccine introduction.

### New TB vaccine policy scenarios

Using the calibrated *no-new-vaccine scenario* model, we projected the impact of *Basecase* scenarios of the introduction of a new vaccine between 2030 and 2050, with vaccine characteristics informed by the M72/AS01_E_ phase IIb trial [11] and expert opinion [30] (Table 1). We assumed all vaccines had 50% efficacy to prevent disease (using different disease definitions as described below) if given to infected or uninfected individuals at the time of vaccination but not to individuals with nTB, aTB, or sTB (i.e., an ‘any infection’ [AI] vaccine [13]), and the duration of protection was 10 years on average with exponential waning, aligned with WHO Preferred Product Characteristics for New Tuberculosis Vaccines [11,31]. To illustrate the potential epidemiologic impact, we assumed a supply and resource-unconstrained maximal rollout of the vaccines. We assumed all vaccines would be delivered routinely to those aged 15 (reaching 80% coverage linearly over 5 years) starting in 2030, and as two campaigns for ages 16–44 (reaching 70% coverage linearly over 5 years) in 2030 and 2040. See S1 Text section 4.2 for full details. Trends in the proportion of the population in each TB state as well as the proportion vaccinated over time are shown in Figure H in S1 Text and Figure I in S1 Text.

**Table 1:** New TB vaccine scenario characteristics.

Characteristics	Prevents progression to infectious symptomatic disease only	Prevents progression to any infectious disease	Prevents progression to any disease
***Policy Scenarios***			
Age targeting	Routine for ages 15, campaign for ages 16–44		
Introduction year (year of repeat campaign)	2030 (2040)		
Achieved coverage	Routine = 80%; Campaign = 70%		
***Vaccine Characteristics by Infection Status and Infectiousness Scenarios***			
Efficacy	50% Sensitivity: 25% or 75%	50%	50% Sensitivity: 25% or 75%
Mechanism of action	Prevents infectious symptomatic disease only	Prevents any infectious disease	Prevents any disease
Host infection status at the time of vaccination required for efficacy	AI Sensitivity: CI or AI + nTB + aTB	AI Sensitivity: CI or AI + nTB	AI Sensitivity: CI
Duration of protection	10 years Sensitivity: 5 or 20 years	10 years	10 years Sensitivity: 5 or 20 years
Relative infectiousness of asymptomatic TB	0.62 to 1 Sensitivity: Low = 0.62 to 0.74, Medium = 0.74 to 0.87, High = 0.87 to 1 and Zero = 0		

Abbreviations: AI, any infection; CI, current infection; nTB, non-infectious TB; aTB, infectious asymptomatic TB.

We simulated three ways the vaccine could affect progression to disease (i.e., the mechanisms of action) [13]:

1. Preventing progression to *infectious symptomatic disease only* (red dashed line in Fig 1B).
2. Preventing progression to *any infectious disease* (blue dash-dotted lines in Fig 1B).
3. Preventing progression to *any disease* (grey dashed lines in Fig 1B).

In this study, we assumed that each vaccine scenario is implemented independently, and that vaccine protection assumed in each scenario was only applied on the relevant indicated flows in Fig 1B. Within each scenario, if an individual did transition across one of the “protected” flows while protected by the vaccine, they would instantly wane their vaccine protection.

### Sensitivity analyses

We analysed the sensitivity of our results to (a) the host infection status at time of vaccination required for efficacy, (b) the relative infectiousness of aTB, (c) whether the vaccine also protects if given to individuals with disease at the time of vaccination, (d) varying vaccine efficacy and (e) varying duration of protection. For (a), we instead assumed that only individuals with infection at time of vaccination could be protected (i.e., modelling a ‘current infection’ vaccine), aligning with the population for which there is evidence for protection from the M72/AS01_E_ Phase 2b trial [13]. For (b), four scenarios of the relative infectiousness were modelled: three scenarios created by dividing the full range of relative infectiousness into low (0.62–0.74), medium (0.74–0.87), and high (0.87–1) categories, and a scenario with zero infectiousness, and the model was recalibrated. For (c), we instead assumed vaccines were also effective in those with pre-symptomatic disease at time of vaccination (nTB and/or aTB in Fig 1C). For (d), we instead assumed the vaccine efficacy was 25% or 75% for vaccines preventing progression to infectious symptomatic disease only or for vaccines preventing progression to any disease, compared to 50% efficacy for vaccines preventing progression to any infectious disease. For (e), we instead assumed the duration of protection was 5 or 20 years on average for vaccines preventing progression to infectious symptomatic disease only or for vaccines preventing progression to any disease, compared to 10 years duration of protection for vaccines preventing progression to any infectious disease. See S1 Text for full details.

### Outcomes

We calculated the number and proportion of sTB, aTB, and nTB episodes averted (where “episodes” represents a single transition into the respective TB disease states), as well as the number and proportion of TB deaths averted between 2030 and 2050. We show the short-term impact between 2030 and 2032 which primarily captures the vaccine direct effects and is a rough approximation to the duration of a typical TB vaccine trial, and the longer-term impact which captures the direct and indirect effects over 20 years.

## Results

### No-new-vaccine scenario results

The *no-new-vaccine* scenario fit all 14 calibration targets with 500 parameter sets (Table E in S1 Text). Over 2030–2050, the *no-new-vaccine* scenario predicted 38.1 million (95% uncertainty interval [UI] = 32.1, 48.4) incident sTB episodes, 95.4 million (UI = 72.7, 123.2) incident aTB episodes, 115.0 million (UI = 67.1, 166.1) incident nTB episodes, and 6.4 million (UI = 5.4, 7.7) TB deaths (Figure E in S1 Text).

### New TB vaccine policy scenario results

**Fig 2:**
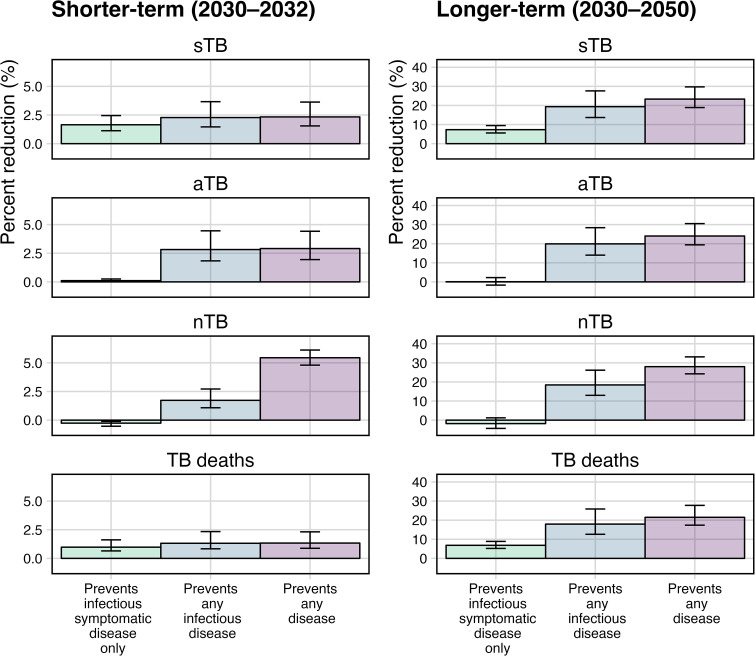
Percent reduction in TB disease episodes and TB deaths by vaccine scenario compared to the no-new-vaccine scenario. Percentage reduction in infectious symptomatic TB episodes (sTB, top row), infectious asymptomatic TB episodes (aTB, second row), non-infectious TB episodes (nTB, third row), and TB deaths (bottom row) over short- (2030–2032, left column) and longer-term (2030–2050, right column), by vaccine disease prevention mechanism of action (preventing infectious symptomatic disease only, any infectious disease, or any disease). Note y-axis scales differ between short and longer term. Error bars represent 95% uncertainty intervals.

The short- (2030–2032) and longer-term (2030–2050) impact of vaccines with 50% efficacy to prevent disease delivered routinely to 15-year-olds and as two campaigns to those aged 16–44 and effective with any infection status at the time of delivery (the *Basecase* scenarios) are shown in Fig 2 (percent averted) and Table 2 (percent and numbers averted).

**Table 2:** Number and cumulative percentage of infectious symptomatic TB (sTB), infectious asymptomatic TB (aTB), infectious symptomatic and infectious asymptomatic TB (aTB + sTB), non-infectious TB (nTB) episodes, and TB deaths averted over short- (2030–2023) and longer-term (2030–2050), by vaccine disease prevention mechanism of action (infectious symptomatic disease only, any infectious disease or any disease).

		Short-term (2030–2032)				Longer-term (2030–2050)			
Vaccine prevents progression to>>		No-new-vaccine scenario	Infectious symptomatic disease only	Any infectious disease	Any disease	No-new-vaccine scenario	Infectious symptomatic disease only	Any infectious disease	Any Disease
**Cumulative numbers, millions (95% UI)**	sTB	6.54 (5.71, 7.92)	6.44 (5.61, 7.77)	6.39 (5.57, 7.71)	6.39 (5.57, 7.70)	38.07 (32.08, 48.36)	35.24 (29.88, 44.5)	30.77 (25.90, 38.34)	29.04 (24.65, 36.08)
	aTB	16.29 (12.85, 20.38)	16.26 (12.84, 20.36)	15.87 (12.47, 19.91)	15.86 (12.46, 19.89)	95.44 (72.72, 123.17)	95.05 (72.83, 122.10)	75.78 (58.16, 98.82)	72.16 (55.39, 92.12)
	aTB + sTB	22.84 (18.63, 27.84)	22.72 (18.48, 27.69)	22.24 (18.07, 27.26)	22.22 (18.06, 27.21)	133.79 (104.56, 169.81)	130.27 (102.14, 164.60)	106.68 (84.37, 135.45)	101.36 (79.71, 127.13)
	nTB	19.56 (12.14, 27.77)	19.63 (12.21, 27.80)	19.23 (11.82, 27.40)	18.41 (11.48, 26.29)	115.00 (67.14, 166.13)	117.48 (70.01, 165.24)	93.71 (53.78, 137.25)	82.03 (48.39, 118.62)
	TB deaths	1.10 (0.97, 1.27)	1.09 (0.96, 1.26)	1.08 (0.96, 1.25)	1.08 (0.96, 1.25)	6.40 (5.36, 7.74)	5.97 (5.00, 7.17)	5.25 (4.37, 6.31)	5.00 (4.20, 6.00)
**Numbers averted, millions (95% UI)**	sTB	–	0.11 (0.07, 0.19)	0.15 (0.09, 0.28)	0.15 (0.1, 0.27)	–	2.80 (1.88, 4.41)	7.27 (4.87, 12.95)	9.01 (6.69, 13.93)
	aTB	–	0.02 (0.01, 0.04)	0.46 (0.28, 0.79)	0.47 (0.3, 0.78)	–	0.09 (−1.46, 2.64)	18.73 (11.9, 31.27)	23.21 (16.33, 33.63)
	aTB + sTB	–	0.13 (0.08, 0.23)	0.61 (0.37, 1.07)	0.62 (0.39, 1.06)	–	2.95 (0.91, 6.59)	25.99 (16.86, 43.78)	32.30 (22.89, 47.11)
	nTB	–	−0.05 (−0.09, −0.03)	0.33 (0.19, 0.49)	1.09 (0.66, 1.52)	–	−2.15 (−4.56, 1.91)	21.32 (12.68, 30.67)	33.23 (18.71, 47.14)
	TB deaths	–	0.01 (0.01, 0.02)	0.01 (0.01, 0.03)	0.01 (0.01, 0.03)	–	0.44 (0.29, 0.64)	1.11 (0.75, 1.84)	1.38 (1.03, 2.00)
**Percent reduction, %** **(95% UI)**	sTB	–	1.65 (1.13, 2.45)	2.28 (1.46, 3.66)	2.33 (1.55, 3.63)	–	7.32 (5.59, 9.48)	19.36 (13.69, 27.63)	23.33 (18.91, 29.72)
	aTB	–	0.11 (0.07, 0.25)	2.82 (1.83, 4.46)	2.91 (1.95, 4.42)	–	0.09 (−1.69, 2.25)	19.90 (14.01, 28.38)	24.02 (19.39, 30.51)
	aTB + sTB	–	0.55 (0.37, 0.91)	2.67 (1.74, 4.21)	2.75 (1.84, 4.18)	–	2.15 (0.72, 4.22)	19.74 (13.92, 28.16)	23.82 (19.25, 30.27)
	nTB	–	−0.26 (−0.53, −0.11)	1.73 (1.08, 2.72)	5.44 (4.80, 6.12)	–	−1.81 (−4.34, 1.20)	18.42 (12.98, 26.15)	27.99 (24.23, 33.12)
	TB deaths	–	0.98 (0.65, 1.62)	1.32 (0.84, 2.34)	1.34 (0.88, 2.31)	–	6.83 (5.20, 8.88)	17.94 (12.61, 25.85)	21.50 (17.39, 27.75)

### Impact on infectious symptomatic TB episodes (sTB)

In the short-term, there was little difference in the proportion of cumulative sTB episodes between vaccines preventing progression to only infectious symptomatic disease, any infectious disease or any disease (1.6% [UI = 1.1, 2.5], 2.3% [UI = 1.5, 3.7] and 2.3% [UI = 1.5, 3.6], respectively) compared to the *no-new-vaccine* scenario (Fig 2, Table 2).

However, over the longer-term, compared to a vaccine that prevented only infectious symptomatic disease, vaccines preventing progression to any infectious disease or any disease averted a much larger proportion of sTB episodes (7.3% [UI = 5.6, 9.5], 19.4% [UI = 13.7, 27.6], and 23.3% [UI = 18.9, 29.7], respectively) (Fig 2, Table 2), due to preventing continued transmission from aTB.

### Impact on infectious asymptomatic TB episodes (aTB)

In the short-term, all three vaccine types averted aTB episodes compared to the *no-new-vaccine* scenario, with reductions of 0.1% (UI = 0.1, 0.3), 2.8% (UI = 1.8, 4.5), and 2.9% (UI = 1.9, 4.4) for vaccines preventing infectious symptomatic disease only, any infectious disease, and any disease respectively (Fig 2, Table 2).

Over the longer-term, only vaccines preventing any infectious disease or any disease averted episodes of aTB compared to the *no-new-vaccine* scenario (19.9% [UI = 14.0, 28.4] and 24.0% [UI = 19.4, 30.5], respectively), whilst there was no impact (0.1% [UI = −1.7, 2.3]) on aTB episodes for a vaccine preventing infectious symptomatic disease only compared to the *no-new-vaccine* scenario (Fig 2, Table 2). The lack of impact on preventing aTB episodes for a vaccine only preventing infectious symptomatic TB was observed due to the accumulation of individuals in aTB contributing to transmission.

### Impact on non-infectious TB episodes (nTB)

In the short-term, there was a small (0.3% [UI = 0.1, 0.5]) *increase* in nTB episodes for a vaccine that prevented infectious symptomatic disease only, and vaccines that prevented any infectious disease or any disease averted 1.7 (UI = 1.1, 2.7) and 5.4% (UI = 4.8, 6.1) of nTB episodes, respectively, compared to the *no-new-vaccine* scenario (Fig 2, Table 2).

Over the longer-term, vaccines that prevented any infectious disease or any disease averted 18.4% (UI = 13.0, 26.1) and 28.0% (UI = 24.2, 33.1) and of nTB episodes, respectively, compared to the *no-new-vaccine* scenario, whilst there was a small (1.8% [UI = −1.2, 4.3]) *increase* in nTB episodes for a vaccine that only prevented infectious symptomatic disease (Fig 2, Table 2). This is because the vaccine only preventing infectious symptomatic disease allows individuals to accumulate and remain in the aTB stage for longer, contributing to greater transmission overall and increasing the number of nTB episodes observed.

### Impact on TB deaths

In the short-term, there was little difference in the proportion of cumulative TB deaths averted by vaccines that prevented only infectious symptomatic disease, any infectious disease, or any disease (1.0% [UI = 0.6, 1.6], 1.3% [UI = 0.8, 2.3], and 1.3% [UI = 0.9, 2.3], respectively).

However, over the longer-term, compared to a vaccine that prevented only infectious symptomatic TB, vaccines preventing any infectious disease or any disease averted a much larger proportion of cumulative TB deaths (6.8% [UI = 5.2, 8.9], 17.9% [UI = 12.6, 25.9], and 21.5% [UI = 17.4, 27.7], respectively) (Fig 2, Table 2), again due to the reduction of transmission by preventing aTB episodes.

### Trends over time

**Fig 3:**
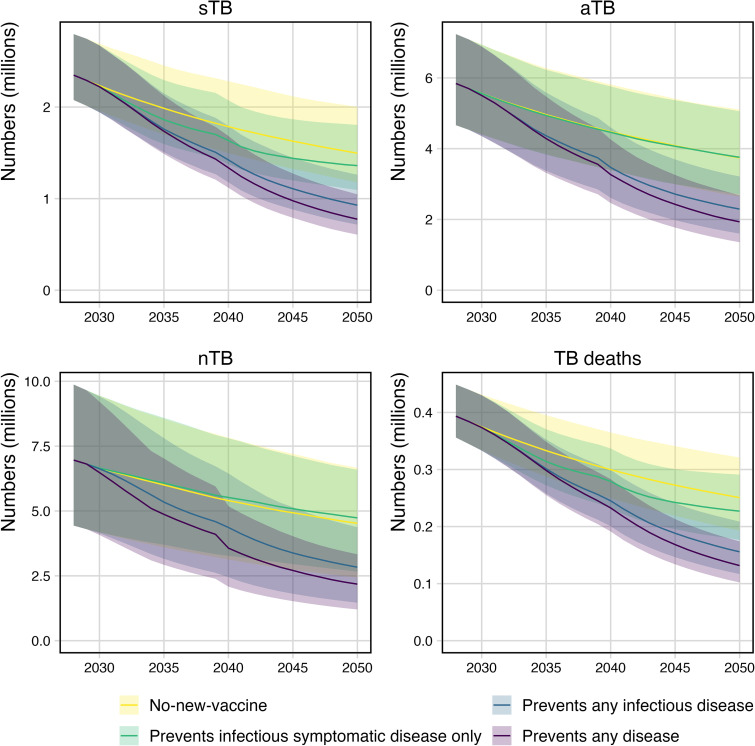
Trends in the number of sTB episodes, aTB episodes, nTB episodes and TB deaths for each vaccine scenario compared to the no-new-vaccine scenario. *Note y-axis scales differ. Shaded areas represent 95% uncertainty intervals*.

Fig 3 shows trends in the number of sTB episodes, aTB episodes, nTB episodes, and TB deaths for the *no-new-vaccine* and each *Basecase* scenario over time from 2030 to 2050. The figure shows that a vaccine preventing only infectious symptomatic disease may *increase* the number of nTB and aTB episodes compared to the *no-new-vaccine* scenario (green trend line versus yellow trend line). A vaccine preventing only infectious symptomatic disease may initially have marked impact on sTB episodes and TB deaths, but this impact may reduce over time as the individuals with aTB accumulate and continue to transmit.

### Sensitivity analyses

#### Efficacy by host infection status at the time of vaccination required for vaccine efficacy.

If the vaccine was only effective in those with current infection at the time of vaccination, as expected, we saw lower impact from all vaccines and at all time points compared to the *Basecase* scenarios. However, the patterns and trends with respect to the mechanism of action of the vaccine were similar to those observed for the *Basecase* scenarios (Figure K, Figure L, and Table H in S1 Text).

### Relative infectiousness of aTB

When the relative infectiousness of aTB compared to sTB was varied within the range of current literature estimates (0.62–1), short- and longer-term results were similar to the *Basecase* scenario impact estimates (Figure M, Table I, Figure N, and Table J in S1 Text). Under the assumption that aTB was not infectious at all, results remained similar to the *Basecase* scenarios in the short-term but differed in the longer-term (Figure M, Table I, Figure N, and Table J in S1 Text) because there was no indirect transmission effect of preventing aTB episodes.

### Efficacy if given to individuals with nTB or aTB

Figure O and Table K in S1 Text show the short-term versus longer-term vaccine impact if an ‘any infection’ or ‘current infection’ vaccine was also effective in early disease stages. Results suggest a vaccine that prevented any infectious disease had greater impact for all outcomes, both short- and longer-term, when effective in individuals with nTB at vaccination, compared to when the vaccine was ineffective if given to those individuals. In contrast, a vaccine that prevented only infectious symptomatic disease had a more pronounced impact when effective in individuals with nTB or aTB, leading to more nTB episodes and greater reductions in sTB episodes and deaths, compared to when ineffective in individuals with nTB or aTB.

### Varying vaccine efficacy and duration of protection

Figure P in S1 Text shows the impact comparing scenarios with varying vaccine efficacy, either decreasing or increasing, and Figure Q in S1 Text shows the impact comparing scenarios with varying duration of protection, either decreasing or increasing. Our findings suggest that the results are robust to uncertainty in vaccine efficacy and duration of protection, and the largest impact over time was still observed for scenarios where aTB was prevented.

## Discussion

This modelling study investigated the sensitivity of impact estimates of new TB vaccines to uncertainties in vaccine efficacy by TB disease stage and aTB infectiousness.

Results suggest that over a three-year period—similar to the duration of a typical vaccine trial—vaccines that prevented only infectious symptomatic disease, any infectious disease, or any form of TB disease averted a similar proportion of cumulative sTB episodes. However, over a 20-year period, which better reflects the full public health impact of vaccination, the differences became more pronounced. Compared to a vaccine that only prevented infectious symptomatic TB, vaccines preventing any infectious disease or any disease averted a significantly higher proportion of sTB episodes. This increased impact was due to preventing the accumulation of and ongoing transmission from aTB. Although the effect was small, a vaccine targeting only infectious symptomatic TB also led to an *increase* in aTB and nTB episodes. These findings highlight the vaccine’s mechanism of action: by preventing only infectious symptomatic disease, this resulted in a pool of individuals with aTB greater than in the no-new-vaccine baseline, sustaining transmission over time. However, this may be reduced with interventions targeting aTB directly.

We explored how the vaccine impact could be affected if we were wrong in some of our key assumptions. In general, our results were robust to our assumptions, but they were sensitive to zero infectiousness of aTB, and if vaccine efficacy was assumed in early pre-symptomatic disease stages. If aTB was assumed to have zero infectiousness, then the differences in impact between vaccine types on preventing sTB episodes was reduced. Over the longer-term, all vaccine types averted a similar proportion of sTB episodes, as accumulation of individuals in the aTB stage under the scenario where the vaccine only prevented progression to symptomatic disease would not result in continued transmission from aTB.

However, in support of the assumption used in the *Basecase* analyses that the infectiousness of aTB relative to sTB was 0.62–1, although limited in number, studies have suggested that aTB *is* likely to be infectious and contribute to transmission [2,32]. We also evaluated scenarios where we assumed, hypothetically, that the vaccines would be effective in pre-symptomatic disease stages at the time of vaccination. If this was the case, we saw greater impact from the vaccines due to protecting a larger proportion of the population, specifically those who were at a high risk of progressing to further disease stages. However, experts believe that it is unlikely that a vaccine would be effective if delivered to someone with disease at the time of vaccination, as the immune response would overwhelm any vaccine effect. Although, whether the same applies for earlier disease stages (such as nTB) and undulation between disease stages is not yet known.

Our work has limitations. We modelled the impact of vaccine scenarios designed to prevent infectious symptomatic disease only, any infectious disease, or any disease independently. We did not assume in this work that there was a scenario which reduced progression from each disease stage to the next for the same individual, although this is being investigated in ongoing work. Given this work was focussed on vaccines that prevented disease, and aligning with the primary endpoints of vaccines currently in trials, we did not investigate vaccines that prevent *Mtb* infection or reinfection, which may also be impactful in reducing the burden of TB in high transmission settings. There remain unknowns with exactly how the mechanism of vaccine protection acts for TB vaccine candidates, which is why this study investigated the impact observed through various potential pathways to prevent disease. Primary estimates of vaccine efficacy and duration of protection were based on trial data and expert input, which may not reflect realised efficacy.

We focussed on several aspects in sensitivity analyses, such as the relative infectiousness of aTB compared to sTB, the host infection status at the time of vaccination required for efficacy, scenarios varying the mechanism of effect, and scenarios with varying vaccine efficacy and duration of protection. We did not investigate scenarios with different levels of vaccine coverage or different delivery strategies. We recognise that the scenarios modelled do not represent an exhaustive list of potential mechanisms of effect of the vaccine, and that additional pathways of protection, such as slowing overall disease progression across the spectrum and the implications for bacterial load, would be important to evaluate, but were outside the scope of this initial piece of work, and are topics for further research.

We also observed a slight increase in the impact of a vaccine that prevents any TB compared to a vaccine that prevents infectious TB. This is because we assumed the vaccine scenario preventing progression to any disease reduces the rate of flow into the nTB compartment and also reduces the rate of flow for those transitions which progress directly from an infection state into aTB. By reducing the rate of flow into nTB in addition to reducing rates directly from infection to aTB, this offers an earlier opportunity to avert progression to infectious TB and increased opportunities to transition to other natural history states, including clearing infection, which reduces the overall risk of subsequent progression into aTB. If our assumptions about the underlying model dynamics or mechanism of action of the vaccine were incorrect, we may have overestimated the impact from a vaccine preventing any disease or underestimated the impact if vaccine protection was retained.

These scenarios were modelled in India, which has the largest burden of TB globally and where large efforts for elimination are ongoing. Its high TB burden, capacity to develop and test vaccines, and the likelihood of early adoption of new TB vaccines, make India a suitable population for this research question. However, these findings may differ in other settings, such as countries with a low burden of TB, different trends in underlying TB dynamics, or differences in the prevalence of comorbidities. We did not account for the effect of comorbidities such as human immunodeficiency virus (HIV), undernutrition, or diabetes on disease transmission and progression. If there is differential impact of a vaccine in people living with HIV or other comorbidities, but much of the TB burden in a country is in that population, then the findings shown here may not hold.

We assumed that coverage of non-vaccine interventions post-2022 would remain constant. If future non-vaccine interventions were scaled up, we will have overestimated absolute vaccine impacts, but relative impacts may be similar. We took a maximalist posture on scale-up of vaccine coverage to illustrate the potential impact, but real-world supply constraints and introduction decisions would likely mitigate the realised impact. However, in support of the values used, a recently published letter from Nelson and colleagues evaluating TB vaccine acceptability in Mozambique provides evidence that these optimistic coverage estimates may be aligned with willingness to accept new TB vaccines in a high TB burden setting, with 77% of participants indicating willingness to receive a new vaccine [33].

Our results depend on natural history structures and initial parameter ranges from established literature reviews, which were consistently reviewed and updated as new data became available. However, given the complexity of the underlying natural history of TB, many of these parameters remain assumptions, and therefore may not accurately represent the dynamics of TB disease. With uncertainty in the inputs, it is important to be conscious that the uncertainty propagates through to the outputs, which is why our modelling results are presented as estimates using numerous fitted parameter sets representing the wide range of assumptions.

We acknowledge that the model structure and dynamics presented here do not perfectly match to what is observed in the real world. For example, we do not capture notified bacteriologically-negative sTB, or possible and treatment of aTB, and these simplifications may lead to underestimates or overestimates in estimated vaccine impact. While the addition of regression from disease states to the natural history structure aligns with updated knowledge of TB dynamics, the model does not have the functionality to track history and quantify “first time episodes” of disease states, which may have resulted in an overestimation of the number of incident episodes averted. We also acknowledge that a symptom-agnostic model could have been set up instead classifying by disease severity. However, this model would likely reach the same qualitative conclusions: a vaccine that does not prevent less severe disease would likely be less valuable, and measuring impact on less severe disease is likely to be important.

Finally, certain modelling assumptions, such as the exclusion of mortality from nTB, represent further simplifications that may impact our projections. As nTB may represent individuals with extra-pulmonary TB, by assuming no extra nTB mortality, we may be underestimating the benefits of early TB stage vaccine efficacy [34] or underestimating the negative impact of individuals accumulating in the nTB and aTB stages. We also assumed that detection and treatment would only occur from the sTB stage, whereas in reality, contact investigation or screening could detect and treat some individuals in the nTB or aTB stages, which could reduce the accumulation of individuals in those stages. These limitations could be investigated in future work.

There are currently around 15 vaccine candidates in trials and the average follow-up time for prevention of disease trial participants is around three years [35]. The primary endpoint of these trials is typically sTB, with impact on aTB either not being measured, or being measured as a secondary and potentially underpowered endpoint. Therefore, at the time of licensure, we may only have powered estimates on the impact of new vaccines on sTB. By having trial data only on the short-term impact on sTB episodes, we will be unable to determine which type of TB disease the vaccine prevents and will not have the data needed to estimate the full public health value of the vaccine. One solution could be to carry out Phase 4 studies after licensure, which may help us know if the wider population level impact is lower than expected. However, this would take many years after licensure for results and would be confounded by other factors such as vaccine coverage and the roll-out of other non-vaccine strategies. Alternatively, a quicker and more robust solution could be to separately measure impact on both aTB and sTB during Phase 2b and 3 trials. We understand that efforts are underway to measure impact on aTB in both the MTBVAC Phase 2b trial (NCT06272812) and M72/AS01_E_ Phase 3 trial (NCT06062238).

If the effect on aTB was measured, for example as a secondary endpoint, by collecting sputum on all participants during or at the end of trials and analysing it at the end of the trial [5], impact on aTB and sTB could be known alongside vaccine licensure and would provide critical evidence on the vaccine mechanism of action for global and country vaccine decision makers considering vaccine roll out. However, as discussed in (Churchyard and colleagues) this has logistical and ethical challenges. Further, by including aTB in the primary endpoint in trials alongside sTB, there is potential for an increase in the number of primary endpoints, which could result in trials being both smaller and shorter [36]. Regardless, the management of individuals with detected aTB must be considered. As it is likely that not all aTB progresses to sTB, if detection of aTB requires treatment, this could expose individuals to unnecessary drug side effects. Alongside sputum collection, it would be useful for future trials to record disease severity to obtain evidence on whether vaccines prevent progression or reduce symptoms.

In addition, empirical data should be collected to better characterise aTB. There are currently limited direct evidence on morbidity, infectiousness, and progression to sTB [36,37]. Future data collection could not only help to better estimate the potential full value of new TB vaccines, but also to better understand the clinical and public health consequences of aTB.

If the assumptions on the characteristics of aTB hold, information from this study may be important. WHO may revise their Preferred Product Characteristics for New TB Vaccines [31] to specify that vaccines should also prevent earlier stages of disease. Vaccine developers may prioritise development of vaccines that also prevent earlier stages of TB disease. TB vaccine trialists may want to test efficacy against aTB to provide timely information about the mechanism of action [5]. It is important to note that a vaccine that only prevents infectious symptomatic disease is still likely to have a large public health benefit on TB morbidity and mortality. However, in addition to vaccine rollout, other programmatic efforts, such as scaled-up active case-finding and treatment for aTB, may be needed to avoid the accumulation of and transmission from individuals with infectious asymptomatic disease.

The Full Value of Vaccine Assessment framework incorporates a wide range of outcomes, both health and non-health (societal/economic), direct and indirect, and to the individual or the population [38]. This study focussed on the traditional direct health impacts of introducing new TB vaccines, but future work could investigate the wider economic impacts of vaccines with varying efficacy by disease stage and estimate whether vaccines that prevent only infectious symptomatic disease could be cost-effective given reductions in TB deaths, treatment costs and loss of productivity.

With our evolving understanding of TB natural history and WHO recognising the potential importance of aTB, we should be open to course-correct, from trial design to estimates of vaccine impact. The population impact of new TB vaccines may depend on efficacy against infectious asymptomatic TB, but whether the difference in impact is large enough to change policy at global or country level will be decided by WHO and national immunisation technical advisory groups (NITAGs). Nevertheless, our results suggest the effect of efficacy against aTB could be significant and supports TB vaccine trials to measure impact on aTB to enable better estimates of the potential full value of new TB vaccines. Further data collection is also required to better understand the transmissibility, morbidity, and dynamics of asymptomatic disease.

## Supporting information

S1 Text **Table A.** India national model parameter values and sources. **Table B.** How age varying parameters are operationalised. **Table C.** Calculating treatment outcome parameter values for adults and children. **Table D.** Calculation of treatment outcomes for India by year. **Table E.** India national model calibration targets. **Table F.** Varying vaccine characteristics in the main and sensitivity analyses. **Table G.** No-new-vaccine baseline incidence of TB episodes and deaths (millions), under baseline and zero aTB infectiousness relative to sTB. **Table H.** Number and percentage cumulative TB episodes and deaths averted (2030–2032 and 2030–2050) for vaccines effective with current infection under baseline aTB infectiousness. **Table I.** Number and percentage cumulative TB episodes and deaths averted between 2030 and 2032 for vaccines effective in any infection, under low, medium, high and zero asymptomatic TB infectiousness relative to symptomatic TB. **Table J.** Number and percentage cumulative TB episodes and deaths averted between 2030 and 2050 for vaccines effective in any infection, under low, medium, high and zero asymptomatic TB infectiousness relative to symptomatic TB. **Table K.** Number and percentage cumulative TB episodes and deaths averted (2030–2032 and 2030–2050) for vaccines effective in any infection or current infection, including disease stages, under baseline asymptomatic TB infectiousness relative to symptomatic TB. **Figure A.** Tuberculosis natural history model structure. **Figure B.** Vaccine structure for an AI or CI vaccine. **Figure C.** TB natural history structure indicating where vaccine efficacy is applied. **Figure D.** TB natural history structure indicating host infection status required for efficacy. **Figure E.** Trends in TB epidemiology from 2005 to 2050 for all ages for model calibrations under varying assumptions about relative asymptomatic TB infectiousness: baseline (0.62, 1), low (0.62, 0.74), medium (0.74, 0.87), and high (0.87, 1). The trend lines in yellow, green, blue and purple indicate the median modelled output with 95% uncertainty reflected by the respective shaded colours. The black dot and vertical lines are the calibration targets from Table E. Note y-axis scales differ. **Figure F.** Trends in TB epidemiology from 2005 to 2050 for all ages for zero relative aTB infectiousness. The black dot and vertical lines are the calibration targets from Table E. Note y-axis scales differ. **Figure G.** Posterior distribution of varying parameters in the baseline relative infectiousness scenario. **Figure H.** Proportion of population vaccinated from 2030 to 2050, for ages 15, 25 and 40 years old. **Figure I.** Proportion in each TB state between 2025 and 2050 across the No-new-vaccine baseline and Basecase scenarios for a randomly selected parameter set. Note y-axis scales differ. *Abbreviations:* aTB, infectious asymptomatic TB; nTB, non-infectious TB; sTB, infectious symptomatic TB. **Figure J.** Prevalence of aTB and sTB between 2025 and 2050 across the No-new-vaccine baseline and Basecase scenarios for a randomly selected parameter set, weighted by the relative infectiousness of aTB. *Abbreviations:* aTB, infectious asymptomatic TB; sTB, infectious symptomatic TB. **Figure K.** Percentage cumulative TB episodes and deaths averted between 2030 and 2032 (left column) or between 2030 and 2050 (right column) for a current infection vaccine. Error bars represent 95% uncertainty intervals. Note y-axis scales differ. *Abbreviations:* aTB, infectious asymptomatic TB; nTB, non-infectious TB; sTB, infectious symptomatic TB. **Figure L.** Trends in the number of sTB, aTB, and nTB episodes and TB deaths between 2030 and 2050 for each current infection vaccine scenario compared to no-new-vaccine scenario for baseline aTB infectiousness. Shaded areas represent 95% uncertainty intervals. Note y-axis scales differ. *Abbreviations:* aTB, infectious asymptomatic TB; nTB, non-infectious TB; sTB, infectious symptomatic TB. **Figure M.** Percentage cumulative TB episodes and deaths averted between 2030 and 2032 for an any infection vaccine by varied relative infectiousness of aTB. Error bars represent 95% uncertainty intervals. *Abbreviations:* aTB, infectious asymptomatic TB; nTB, Non-infectious TB; sTB, infectious symptomatic TB. **Figure N.** Percentage cumulative TB episodes and deaths averted between 2030 and 2050 for an any infection vaccine by varied relative infectiousness of aTB. Error bars represent 95% uncertainty intervals. *Abbreviations:* aTB, infectious asymptomatic TB; nTB, non-infectious TB; sTB, infectious symptomatic TB. **Figure O.** Percentage cumulative TB episodes and deaths averted between 2030 and 2032 (left column) or between 2030 and 2050 (right column), where vaccines were effective with any infection or current infection, including pre-symptomatic disease stages, with baseline (0.62, 1) infectiousness of aTB relative to sTB. Error bars represent 95% uncertainty intervals. Note y-axis scales differ. *Abbreviations:* AI, any infection; aTB, infectious asymptomatic TB; CI, current infection; nTB, non-infectious TB; sTB, infectious symptomatic TB. **Figure P.** Percentage cumulative TB episodes and deaths averted between 2030 and 2032 (left column) or between 2030 and 2050 (right column). Error bars represent 95% uncertainty intervals. Note y-axis scales differ. *Abbreviations:* aTB, infectious asymptomatic TB; nTB, non-infectious TB; sTB, infectious symptomatic TB. **Figure Q.** Percentage cumulative TB episodes and deaths averted between 2030 and 2032 (left column) or between 2030 and 2050 (right column). Error bars represent 95% uncertainty intervals. Note y-axis scales differ. *Abbreviations:* aTB, infectious asymptomatic TB; nTB, non-infectious TB; sTB, infectious symptomatic TB.
(DOCX)

## Abbreviations

aTB: infectious asymptomatic TB
HIV: human immunodeficiency virus
Mtb: Mycobacterium tuberculosis
nTB: non-infectious TB
NITAG: national immunisation technical advisory group
sTB: infectious symptomatic TB
TB: Tuberculosis
UI: uncertainty interval
WHO: World Health Organization
