# Supplementary material for: Estimating the population impact of new tuberculosis vaccines depending on efficacy against infectious asymptomatic tuberculosis: A modelling study
Source: PLoS Med. 2026 Feb 12;23(2):e1004595. doi: 10.1371/journal.pmed.1004595 (PMC12928567; doi:10.1371/journal.pmed.1004595)
Supplement: S1 Text — Table A. India national model parameter values and sources. Table B. How age varying parameters are operationalised. Table C. Calculating treatment outcome parameter values for adults and children. Table D. Calculation of treatment outcomes for India by year. Table E. India national model calibration targets. Table F. Varying vaccine characteristics in the main and sensitivity analyses. Table G. No-new-vaccine baseline incidence of TB episodes and deaths (millions), under baseline and zero aTB infectiousness relative to sTB. Table H. Number and percentage cumulative TB episodes and deaths averted (2030–2032 and 2030–2050) for vaccines effective with current infection under baseline aTB infectiousness. Table I. Number and percentage cumulative TB episodes and deaths averted between 2030 and 2032 for vaccines effective in any infection, under low, medium, high and zero asymptomatic TB infectiousness relative to symptomatic TB. Table J. Number and percentage cumulative TB episodes and deaths averted between 2030 and 2050 for vaccines effective in any infection, under low, medium, high and zero asymptomatic TB infectiousness relative to symptomatic TB. Table K. Number and percentage cumulative TB episodes and deaths averted (2030–2032 and 2030–2050) for vaccines effective in any infection or current infection, including disease stages, under baseline asymptomatic TB infectiousness relative to symptomatic TB. Figure A. Tuberculosis natural history model structure. Figure B. Vaccine structure for an AI or CI vaccine. Figure C. TB natural history structure indicating where vaccine efficacy is applied. Figure D. TB natural history structure indicating host infection status required for efficacy. Figure E. Trends in TB epidemiology from 2005 to 2050 for all ages for model calibrations under varying assumptions about relative asymptomatic TB infectiousness: baseline (0.62, 1), low (0.62, 0.74), medium (0.74, 0.87), and high (0.87, 1). The trend lines in yellow, green, blue an [file pmed.1004595.s001.docx]

***Supplementary material for***

***Estimating the population impact of new tuberculosis vaccines depending on efficacy against infectious asymptomatic tuberculosis: a modelling study***

*Hira Tanvir, Rebecca A Clark, Tom Sumner, Katherine C Horton, Tomos O Prŷs-Jones, Roel Bakker, Kirankumar Rade, Vidya Mave, Mark Hatherill, Gavin Churchyard, Rein MGJ Houben, Richard G White*

**Table of Contents**

SUPPORTING METHODS 1

1. Model structure and equations 1

1.1 Natural history model structure 1

1.2 Natural history scenarios 2

1.3 Model equations 3

2. Model Parameters and Data Sources 4

2.1 Natural history parameter values and data sources 4

2.2 Age varying parameters 7

2.3 Treatment initiation and outcomes 8

3. Model simulation and calibration 10

3.1    Model simulation 10

3.2    Model calibration 10

7. Policy scenarios 12

4.1 No-new-vaccine scenario 12

4.2 Vaccine scenarios 12

Vaccine model structure 15

Vaccine integration in the TB natural history model 16

SUPPORTING RESULTS 18

8. No-new-vaccine scenario results 18

5.1 No-new-vaccine calibration (with baseline, low, medium and high relative infectiousness) 18

5.2 No-new-vaccine scenario calibration (with zero relative infectiousness) 19

9. Vaccinated proportions over time 21

10. Proportion in each TB state over time 22

11. Sensitivity analysis results: vaccines effective with current infection status 24

12. Trends over time for vaccines effective with current infection status 26

13. Sensitivity analysis results: vaccines effective with any infection status with varying infectiousness 27

9.1 Short-term impact (2030–2032) 27

9.2 Longer-term impact (2030–2032) 29

14. Sensitivity analysis results: vaccines effective with any or current infection status including efficacy in pre-disease stages 31

15. Sensitivity analysis results: scenarios with varying vaccine efficacy 34

16. Sensitivity analysis results: scenarios with varying duration of protection 35

# SUPPORTING METHODS

## Model structure and equations

We created an age-stratified compartmental differential equation model of tuberculosis in India, including dimensions for age, tuberculosis natural history, vaccination, and treatment. The age structure is identical to that included in Clark et al., *BMC Medicine*, 2023 (1). The natural history structure is an adaptation from Clark et al. informed by work on the natural history of TB from Richards et al., *Lancet Glob Health*, 2023 (2) and Horton et al., *PNAS*, 2023 (3). Symptomatic disease compartment naming has been done adhering to current TB definitions according to the latest WHO guidance, and definitions of asymptomatic TB following a WHO consultation 2024 (4).

### 1.1 Natural history model structure


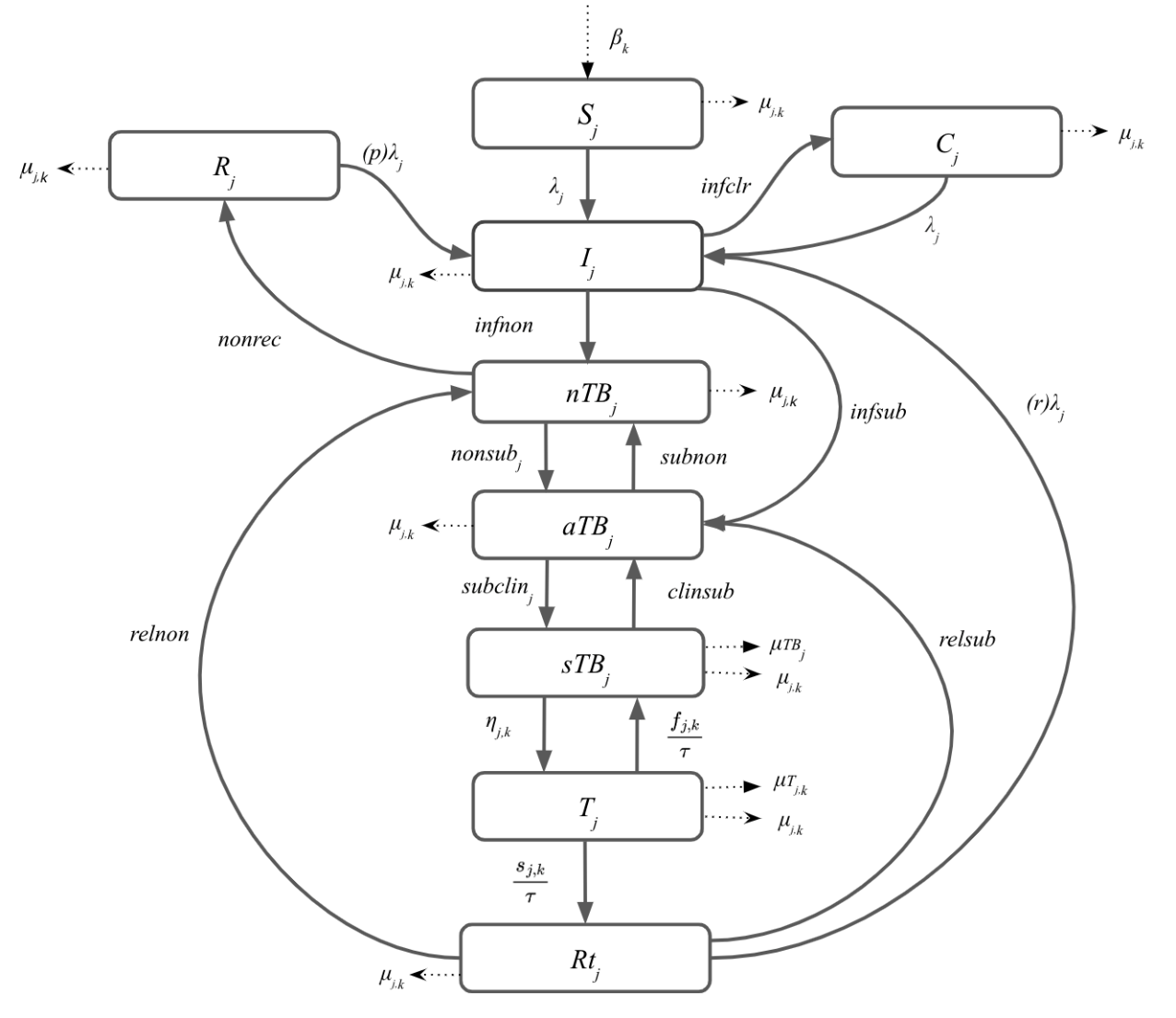


Figure A Tuberculosis natural history model structure

*Abbreviations: S = Susceptible; I = Infection; C = Cleared; nTB = Non-infectious TB; R = Recovered; aTB = Infectious asymptomatic TB; sTB = Infectious symptomatic TB; T = On-Treatment; Rt = Recovered after treatment. Subscript j represents parameters that vary by age, and subscript k represents parameters that vary over time.*

A natural history structure with nine compartments in Figure A was created by adapting features of previous models.

- Susceptible individuals (S) are infected at rate $\lambda$ and progress to the infected compartment. Infected individuals $(I)$ may clear infection $(C)$ at rate $infclr$ or progress to non-infectious disease $(nTB)$or infectious asymptomatic disease $(aTB)$ at rates$infnon$ and $infsub$, respectively.
- Individuals with non-infectious disease $(nTB)$ recover $(R)$ at rate $nonrec$ or progress to infectious asymptomatic disease $(aTB)$ at rate $nonsub$. Individuals with infectious asymptomatic disease $(aTB)$ regress to non-infectious disease $(nTB)$ at rate $subnon$ or progress to infectious symptomatic disease $(sTB)$ at rate $subclin$. Individuals with infectious symptomatic disease $(sTB)$ regress to infectious asymptomatic disease $(aTB)$ at rate $clinsub$ or die from TB-associated mortality at rate$\mu TB$.
- Individuals with infectious symptomatic disease $(sTB)$ progress to on-treatment $(T)$ at rate $\eta$. Individuals on-treatment $(T)$ have three possible treatment outcomes, whereby individuals can progress to recovered after treatment $(Rt)$ at on-treatment completion rate $\frac{s_{j}}{\tau}$, individuals can regress to infectious symptomatic disease $(sTB)$ at on-treatment non-completion rate $\frac{f_{j}}{\tau}$, or die while on-treatment.
- Individuals recovered after treatment $(Rt)$ may relapse to non-infectious disease $(nTB)$ or to infectious asymptomatic disease $(aTB)$ at rates $relnon$ and $relsub$, respectively.
- Individuals who have recovered $(R)$from non-infectious disease $(nTB)$ are protected from reinfection with an effective reinfection rate of $p\lambda$ and those who have recovered after treatment $(Rt)$ have an increased risk of reinfection ($r\lambda)$.

### 1.2 Natural history scenarios

We explored the impact of varying the infectiousness of aTB relative to sTB on the impact of TB vaccines by dividing the baseline range of aTB infectiousness relative to sTB (0.62 to 1) into three equal segments: low (0.62 to 0.74), medium (0.74 to 0.87), and high (0.87 to 1) infectiousness. We then calibrated the model to reflect all four scenarios—baseline, low, medium, and high—and compared the effect of the infectiousness scenarios on the resulting impact of TB vaccines. We also investigated a scenario where relative aTB infectiousness was zero.

### 1.3 Model equations

**Natural history model equations**

$\frac{{dS}_{j}}{dt}= \beta_{k}-\left( \lambda_{j}+\mu_{j,k} \right)S_{j} {where Age}_{j}=0$

$$\frac{{dS}_{j}}{dt}= -\left( \lambda_{j}+\mu_{j,k} \right)S_{j} {where Age}_{j}\neq0$$

$$\frac{{dC}_{j}}{dt}= {infclr(I}_{j})-\left( \lambda_{j}+\mu_{j,k} \right)C_{j}$$

$$\frac{{dR}_{j}}{dt}= {nonrec(nTB}_{j})-\left( {p\lambda}_{j}+\mu_{j,k} \right)R_{j}$$

$$\frac{{dI}_{j}}{dt}= {\lambda_{j}(S}_{j}+C_{j}+pR_{j}+r{Rt}_{j})-\left( infclr+infnon+infsub+\mu_{j,k} \right)I_{j}$$

$$\frac{{dnTB}_{j}}{dt}= infnon{(I}_{j})+relnon\left( {Rt}_{j} \right)+subnon\left( {aTB}_{j} \right)-\left( nonrec+{nonsub}_{j}+\mu_{j,k} \right){nTB}_{j}$$

$$\frac{{daTB}_{j}}{dt}= infsub{(I}_{j})+{nonsub}_{j}\left( {nTB}_{j} \right)+relsub\left( {Rt}_{j} \right)+clinsub\left( {sTB}_{j} \right)- \left( subnon+{subclin}_{j}+\mu_{j,k} \right){aTB}_{j}$$

$$\frac{{dsTB}_{j}}{dt}= {subclin}_{j}\left( {aTB}_{j} \right)+\frac{f_{j,k}}{\tau}\left( T_{j} \right)-\left( clinsub+\eta_{j,k}+\mu_{{TB}_{j}}+\mu_{j,k} \right){sTB}_{j}$$

$$\frac{{dT}_{j}}{dt}= \eta_{j,k}\left( {sTB}_{j} \right)-\left( \frac{s_{j,k}+f_{j,k}}{\tau}+\mu_{T_{j,k}}+\mu_{j,k} \right)T_{j}$$

$$\frac{{dRt}_{j}}{dt}= \frac{s_{j,k}}{\tau}\left( T_{j} \right)-\left( relnon+relsub+r\lambda_{j}+\mu_{j,k} \right){Rt}_{j}$$

**Force of infection equation**

Susceptible individuals (S) are infected at rate $\lambda$. $\lambda$is defined as follows:

$$\lambda_{j}=pT\cdot\sum_{y=1}^{n_{ygroups}} C\left[ m,y \right]\cdot\left( \frac{\left( 1-ep \right)\cdot\left( T_{{sTB}_{y}}+tT_{{aTB}_{y}} \right)\cdot k_{inf}}{N_{y}} \right)$$

$j$ indicates age of individual in years

$pT$ indicates probability of transmission per infectious contact

$n_{groups}$ indicates the number of contact age groups

$C\left[ m,y \right]$ indicates the number of age-specific contacts

$m$ indicates age group of individual

$y$ indicates the age group of contact

$T_{{sTB}_{y}}$ indicates the total number of individuals with symptomatic TB in age group *y*

$T_{{aTB}_{y}}$ indicates the total number of individuals with asymptomatic TB in age group *y*

$k_{inf}$ indicates the relative infectiousness of children (<15 years) relative to adults (≥15 years)

$ep$ indicates the average proportion of TB cases that are extrapulmonary

$t$ indicates the infectiousness of asymptomatic TB relative to symptomatic TB

$N_{y}$ indicates the total population in age group *y*

## Model Parameters and Data Sources

### 2.1 Natural history parameter values and data sources

Table A shows the India model parameters and sources used in the natural history model structure, along with their definitions, sources, and information on whether the parameter was fixed or varied (as well as whether they were varied by age or time) during calibration.

Further details about how the age varying parameters were implemented are provided in section 2.2, and further details related to TB treatment are provided in section 2.3. The parameter ranges provided for the TB natural history parameters are priors used during calibration in a Bayesian analysis. We assumed that all values within the prior range were equally likely. The prior ranges were pre-specified based on literature review and were reviewed as new data became available.

**Table A India national model parameter values and sources**

| **Description** | **Units** | **Symbol** | **Initial Range** | **Fixed or Varying During Calibration** | **Age Varying** | **Time Varying** | **Source** |
| --- | --- | --- | --- | --- | --- | --- | --- |
| ***Births and deaths (excluding on-treatment mortality)*** | | | | | | | |
| Birth rate | Per year | $\beta_{k}$ | UN World Population Prospects population estimates and projections | Fixed | No | Yes | (5) |
| Background mortality rate | Per year | $\mu_{j,k}$ | Calculated in the model from UN population estimates and projections | Fixed | Yes, age specific mortality rates from demographic dataset | Yes | (5) |
| Mortality rate for $sTB$ | Per person  per year | ${\mu TB}_{j}$ | (0, 0.38) | Varying | Yes, value for children is greater than value for adults | No | (3) |
| ***Natural History*** | | | | | | | |
| Force of infection | Per year | $\lambda_{j}$ | Fitted | Fixed Equation | Yes, age specific contact rates(6) | No | *Calculated* |
| Probability of transmission per infectious contact | - | $pT$ | (0, 0.0068) | Varying | No | No | *Assumed* |
| Fraction of total TB that is extrapulmonary | - | $ep$ | 0.1972 | Fixed | No | No | (7,8) |
| Infectiousness of $aTB$ relative to $sTB$ | - | $t$ | (0.62, 1) | Varying | No | No | (9) |
| Rate from $I$ to $aTB$ | Per person per year | $infsub$ | (0.01, 0.1) | Varying | No | No | (3) |
| Rate from $I$ to $nTB$ | Per person per year | $infnon$ | (0.04, 0.23) | Varying | No | No | (3) |
| Rate from $I$to $C$ | Per person per year | $infclr$ | (0.93, 3.30) | Varying | No | No | (3) |
| Rate from $nTB$to $R$ | Per person per year | $nonrec$ | (0.14, 0.23) | Varying | No | No | (3) |
| Rate from $nTB$ to $aTB$ | Per person per year | $nonsub_{j}$ | (0.21, 0.28) | Varying | Yes; value for children   is **less** than value for adults. | No | (3) |
| Rate from $aTB$ to $nTB$ | Per person per year | $subnon$ | (1.24, 2.03) | Varying | No | No | (3) |
| Rate from $aTB$ to $sTB$ | Per person per year | $subclin_{j}$ | (0.08, 2.93) | Varying | Yes; value for children   is **less** than value for adults. | No | (3) |
| Rate from $sTB$ to $aTB$ | Per person per year | $clinsub$ | (0.46, 0.72) | Varying | No | No | (3) |
| Rate of relapse from $Rt$ to $nTB$ | Per person  per year | $relnon$ | (0, 0.01) | Varying | No | No | *Assumed* |
| Rate of relapse from $Rt$to $aTB$ | Per person  per year | $relsub$ | (0, 0.01) | Varying | No | No | *Assumed* |
| ***Protection Parameters*** | | | | | | | |
| Relative risk of re-infection in $R$ | - | $p$ | (0.14, 0.30) | Varying | No | No | (10) |
| Relative risk of re-infection in $Rt$ | - | $r$ | (2.14, 4.27) | Varying | No | No | (11) |
| ***Treatment parameters*** | | | | | | | |
| Treatment initiation from $sTB$ | Per person per year | $\eta_{j,k}$ | (0, 2) | Sigmoidal curve describing rate of treatment initiation | Yes; value for children   is **less** than value for adults. | Yes | *Assumed* |
| Treatment duration | Number of years | $\tau$ | 0.5 | Fixed | No | No | (12,13) |
| Rate of on-treatment mortality | Per person per year | ${\mu T}_{j}=\frac{k_{j}}{\tau}$ | Country-specific | Varying | Yes; value for children  **greater** than value for adults. | Yes | (14) |
| Rate of treatment completion | Per person per year | $\frac{s_{j}}{\tau}$ | Country-specific | Fixed equation | Yes, indirectly scaled by $k_{mort}$ | Yes | (14) |
| Rate of treatment non-completion | Per person per year | $\frac{f_{j}}{\tau}$ | Country-specific | Fixed equation | Yes, indirectly scaled by $k_{mort}$ | Yes | (14) |

### 2.2 Age varying parameters

We assume that aspects of TB natural history and mortality vary by age. This is implemented by stratifying certain natural history parameters by age and applying age-specific prior ranges and relative constraints during calibration (15). The following table describes the method used to operationalise the age varying differences in parameters between adults (ages ≥15 years) and children (ages <15 years). For the rates per year of progression to TB disease, we assumed that the rate for children is less than that for adults. For mortality rates, we assumed the opposite: the rate for children is higher than that for adults.

Table B How age varying parameters are operationalized

| **Parameter** | **Range** | **Age Varying Description** | **Age Scaling Parameter** | **Adults**  **(age 15+)** | **Children**  **(age 0–14)** |
| --- | --- | --- | --- | --- | --- |
| ${nonsub}_{j}$  Rate per year of fast progression from Infection to asymptomatic TB | (0.21, 0.28) | Retain if value for children is less than value for adults | Sample $k_{prog}$  from (0.5, 1) | Sample *nonsub_A15_* from (0.21, 0.28) | $\max\left( 0.21, {nonsub}_{A15}\times k_{prog} \right)$ |
| ${subclin}_{j}$  Rate per year progression from asymptomatic TB disease to symptomatic disease | (0.08, 2.93) | Retain if value for children is less than value for adults | Sample $k_{prog}$  from (0.5, 1) | Sample *subclin_A15_* from (0.08, 2.93) | $\max\left( 0.08, {subclin}_{A15}\times k_{prog} \right)$ |
| $\eta_{j}$  Rate per year of treatment initiation | (0, 2) | Retain if value for children is less than value for adults | Sample $k_{dx}$  from (0.5, 1) | Sample *η_A15_* from (0, 2) | $\max\left( 0, \eta_{A15}\times k_{dx} \right)$ |
| ${\mu TB}_{j}$  Symptomatic TB mortality rate per year | (0, 0.38) | Retain if value for children is greater than value for adults | Sample $k_{mort}$ from  (1, 1.5) | *μTB_A0_* 𝗑 $k_{mort}$ | Sample *μTB_A0_* from  (0, 0.38) |
| ${\mu T}_{j}=\frac{k_{j}}{\tau}$  On-treatment mortality rate per year | $\left( 0, \frac{k_{max}}{\tau} \right)$ | Retain if value for children is greater than value for adults | Sample $k_{mort}$from  (1, 1.5) | $\frac{K_{A0}}{\tau}\times k_{mort}$ | Sample $\kappa_{A0}$ from  (0, 0.135) |

### 2.3 Treatment initiation and outcomes

Steps for calculating treatment initiation, treatment completion, non-completion, and mortality rates are described in section 3 of the Supplementary Material for Clark et al., *Lancet Glob Health*, 2023 and summarised in Table C below (16).

**Table C** **Calculating treatment outcome parameter values for adults and children**

| **Parameter** | **Adults** | **Children** |
| --- | --- | --- |
| $\kappa_{j}$  On-treatment mortality fraction | $\kappa_{A0}\times\kappa_{mort}$ | Sample $\kappa_{A0}$ from $(0, 0.135)$ |
| $s_{j}$  On-treatment completion fraction | $\left( 1-\kappa_{A15} \right)SFR$ | $\left( 1-\kappa_{A0} \right)SFR$ |
| $f_{j}$  On-treatment non-completion fraction | $\left( 1-\kappa_{A15} \right)(1-SFR)$ | $\left( 1-\kappa_{A0} \right)(1-SFR)$ |

SFR is the ratio between treatment completions to the sum of the number of treatment completions and non-completions. In India, SFR = 0.96. The data used to calculate the on-treatment outcomes was obtained from the WHO. However, as the private sector accounts for a substantial portion of treatments in India, and not all the treatments conducted in the private sector are reported, we adjust the on-treatment completion and non-completion fractions from Table C as described below and in Table D. Additionally, each of the parameters in Table C were divided by 𝜏 to obtain the on-treatment mortality rate per year, on-treatment completion rate per year, and on-treatment non-completion rate per year.

As described in Clark et al., *BMC Medicine*, 2023 (included here with minor text modifications), we adjusted the treatment outcomes to account for treatment occurring in both the public and private sectors (1). We assumed that the total number of treatments was composed of the treatments that are reported and the treatments that are not reported. We assumed that 60% of the total treatment occurs in the public sector and the remaining 40% occurred in the private sector. We assumed that all treatments not reported were from the private sector, that the treatment completion rate in the private sector was 40%, and that there was no reporting bias (in that they were equally likely to not report treatment completions, non-completions, or deaths). Before 2012, only the treatment conducted in the public sector was reported, but since then, treatment in the private sector has begun to be reported, which is reflected by the increasing total fraction of treatments reported (17). We assumed that the on-treatment mortality fraction was the same in the public and private sector but adjusted the treatment completion and non-completion rates to account for differences between those reported and those not reported as in Table D.

**Table D** **Calculation of treatment outcomes for India by year**

| **Description** | **Symbol** | **Year** $\boldsymbol{(k)}$ | | | | | | | | |
| --- | --- | --- | --- | --- | --- | --- | --- | --- | --- | --- |
|  |  | **≤2012** | **2013** | **2014** | **2015** | **2016** | **2017** | **2018** | **2019** | **≥2020** |
| Fraction of total treatments reported | $F_{T,k}$ | 0.60 | 0.63 | 0.68 | 0.67 | 0.73 | 0.77 | 0.80 | 0.83 | 0.87 |
| On-treatment mortality rate | $\frac{\kappa_{j}}{\tau}$ | Sample $\kappa_{A0}$ from $(0, 0.135)$ then $\kappa_{A15}=\kappa_{A0}\times\kappa_{mort}$ | | | | | | | | |
| On-treatment completion rate | $\frac{s_{j}}{\tau}$ | $\frac{F_{T,k}0.96\left( 1-\kappa_{j} \right)+\left( 1-F_{T,k} \right)0.40}{\tau}$ | | | | | | | | |
| On-treatment non-completion rate | $\frac{f_{j}}{\tau}$ | $\frac{F_{T,k}0.04\left( 1-\kappa_{j} \right)+\left( 1-F_{T,k} \right)(0.60-\kappa_{j})}{\tau}$ | | | | | | | | |

## Model simulation and calibration

### 3.1    Model simulation

We specified a system of ordinary differential equations defining the derivatives with respect to time of a set of state variables, to simulate the country-specific TB epidemic between 1900 and 2050. We initialised the simulation by distributing the population between the TB natural history states using a fitted parameter representing the proportion of the population uninfected at the start of the simulation. For each year of the simulation (1900–2050), our model is designed to exactly match the age and country-specific UN population estimates and projections. (5)

### 3.2    Model calibration

For this India modelling analysis, we followed the same modelling approach as in Clark et al., *BMC Medicine*, 2023 (1).

Broadly, this was as follows:

1. Construct a mechanistic model
2. Calibrate the model by identifying areas of the input parameter space where the output of the mechanistic model was consistent with the historical epidemiologic data
3. Use the calibrated model to simulate and predict future TB epidemiology and new vaccines

In the context of this analysis, step 1 was achieved by creating the compartment differential equation model as specified in Section 1. For step 2, we independently calibrated a model by identifying areas of the parameter space that made the output of the model match the corresponding calibration targets (from Table E below). The model was fitted to the calibration targets using history matching with emulation, a method that allows us to explore high-dimensional parameter spaces efficiently and robustly (18–20). History matching progresses as a series of iterations, called waves, where implausible areas of the parameter space, i.e., areas that are unable to give a match between the model output (e.g., the predicted incidence rate by the model) and the empirical data (e.g., the incidence rate calibration target from the WHO data), are found and discarded. In order to identify implausible parameter sets, emulators, which are statistical approximations of model outputs that are built using a modest number of model runs, are used. Emulators provide an estimate of the value of the model at any parameter set of interest, with the advantage that they are orders of magnitude faster than the model.

History matching with emulation, implemented through the *hmer* package in R (21), considerably reduced the size of the parameter space to investigate. Rejection sampling was then performed on the reduced space to identify at least 500 parameter sets that matched all targets. Once we had obtained 500 parameter sets that produced output consistent with the calibration targets, we used those parameter sets with the mechanistic model to simulate the future (step 3).

Table E shows the calibration targets for India. Modifications to calibration targets were made as described in the supplementary material (section 3.4) of Clark et al., *BMC Medicine*, 2023 (1). Targets for all ages were the TB incidence rate in 2010 and 2020 (276 [114, 508] and 188 [129, 257] per 100,000 population), mortality rate in 2010 and 2020 (47 [34, 62] and 37 [34, 40] per 100,000 population), case notification rate in 2010 and 2020 (180 [144, 216] and 136 [109, 163] per 100,000 population), infectious TB prevalence in 2015 and 2021 (315 [210, 529] and 312 [218, 406] per 100,000), and aTB prevalence ratio in 2021 (0.564 [0.533, 0.595]) (5,7,8,22–25). Targets for children were the incidence rate and case notification rate in 2020 (91 [56, 126] and 33 [26, 40] per 100,000 population, respectively) (5,8,24). Targets for adults were the TB incidence rate and case notification rate in 2020 (224 [138, 310] and 173 [138, 208] per 100,000 population) and infectious TB prevalence in 2021 (394 [276, 512] per 100,000 population) (5,8,22,24).

Table E India national model calibration targets

| **Calibration Targets** | **Year** | **Age (years)** | **Estimate** | **Lower** | **Upper** |
| --- | --- | --- | --- | --- | --- |
| TB incidence rate (per 100,000 population/year) | 2010(7) | All | 276 | 114 | 508 |
|  | 2020(24) | All | 188 | 129 | 257 |
|  |  | 0–14 | 91 | 56 | 126 |
|  |  | ≥15 | 224 | 138 | 310 |
| TB mortality rate (per 100,000 population/year) | 2010(7) | All | 47 | 34 | 62 |
|  | 2020(7) | All | 37 | 34 | 40 |
| TB case notification rate  (per 100,000 population/year) | 2010(5,8) | All | 180 | 144 | 216 |
|  | 2020(5,8) | All | 136 | 109 | 163 |
|  |  | 0–14 | 33 | 26 | 40 |
|  |  | ≥15 | 173 | 138 | 208 |
| Infectious TB prevalence  (per 100,000 population) | 2015(23,25) | All | 315 | 210 | 529 |
|  | 2021(22) | All | 312 | 218 | 406 |
|  | 2021(22) | ≥15 | 394 | 276 | 512 |
| Asymptomatic-to-symptomatic TB prevalence ratio | 2021(22) | ≥15 | 0.564 | 0.533 | 0.595 |

## Policy scenarios

### 4.1 No-new-vaccine scenario

The no-new-vaccine scenario assumed non-vaccine TB interventions continued at current levels into the future. As reported country-level data includes the high coverage levels of neonatal BCG vaccination, this was not explicitly modelled, and we assumed that BCG vaccination would not be discontinued over the model time horizon. We assumed the infectiousness of $aTB$ relative to $sTB$ in the no-new-vaccine scenario ranged between $(0.62, 1)$. (9)

### 4.2 Vaccine scenarios

***Vaccine eligible population***

We assumed that there was no pre-vaccination infection testing. Therefore, even if a vaccine was only effective when delivered to those with current infection at the time of vaccination, we assumed that all individuals would receive the vaccine, and only those with current infection would receive protection.

***Vaccine efficacy and protection from repeat vaccinations***

Aligning with M72/AS01_E_ Phase 2b trial results which demonstrated 49.7% (95% confidence interval = 2.1, 74.2) efficacy to prevent disease in latently infected adults (26), we assumed 50% prevention of disease efficacy. We assumed that protection increases if a subsequent vaccine course is administered while the individual is currently protected by (1 – current protection) times vaccine efficacy. The number of vaccine courses refers to the number of vaccine courses that the individual is currently protected by, not that they have ever received or have ever been protected by.

***Constant vaccine delivery characteristics***

We assumed that all vaccines had 10-years duration of protection on average with exponential waning. All vaccine scenarios were delivered through routine vaccination of 15-year-olds (80% coverage, aligning with HPV coverage in South Africa combined with aggregated secondary school enrolment in China and India (27,28), as assumed in Harris et al., 2020) starting in 2030, a campaign for ages 16–44 in 2030 (scaled up to 70% coverage over five years, which aligns with the lower bound of the MenAfriVac campaigns in sub-Saharan Africa (29) as also assumed in Harris et al., 2020) and 2040 (reaching 70% coverage over one year).

***Varied vaccine characteristics***

The following vaccine characteristics were varied in this study: Host infection status at the time of vaccination required for vaccine efficacy, mechanism of prevention of disease action, the relative aTB infectiousness, vaccine efficacy, and duration of protection. Table **F** below outlines the key varied vaccine characteristics for the vaccine scenarios used in the main and sensitivity analyses.

**Table F Varying vaccine characteristics in the main and sensitivity analyses**

|  | **Characteristic** | | | | | | |
| --- | --- | --- | --- | --- | --- | --- | --- |
|  | **Host stages at the time of vaccination required for vaccine efficacy** | **Mechanism of prevention of disease action** | **Waning conditions** | **Relative aTB infectiousness** | | **Efficacy** | **Duration of protection** |
| ***Main analyses*** | | | | | | | |
| Prevention of only infectious symptomatic disease | AI excluding disease | Reduces progression from aTB to sTB via *subclin* | Efficacy lost once individuals progress into sTB | 0.62, 1 | | 50% | 10 years |
| Prevention of any infectious disease | AI excluding disease | Reduces progression to aTB from I and nTB via *nonsub*, *infsub* and *relsub*. | Efficacy lost once individuals progress into aTB | 0.62, 1 | | 50% | 10 years |
| Prevention of any disease | AI excluding disease | Reduces progression from infection to nTB/aTB via *infnon*, *infsub*, *relnon*, and *relsub*. | Efficacy lost once individuals progress into nTB/aTB | 0.62, 1 | | 50% | 10 years |
| ***Sensitivity analyses*** | | | | | | | |
| Prevention of only infectious symptomatic disease | CI excluding disease | Reduces progression from aTB to sTB via *subclin* | Efficacy lost once individuals progress into sTB | 0.62, 1 | | 50% | 10 years |
| Prevention of any infectious disease | CI excluding disease | Reduces progression to aTB from I and nTB via *nonsub*, *infsub* and *relsub*. | Efficacy lost once individuals progress into aTB | 0.62, 1 | | 50% | 10 years |
| Prevention of any disease | CI excluding disease | Reduces progression from infection to nTB/aTB via *infnon*, *infsub*, *relnon*, and *relsub*. | Efficacy lost once individuals progress into nTB/aTB | 0.62, 1 | | 50% | 10 years |
| Prevention of only infectious symptomatic disease | AI excluding disease | Reduces progression from aTB to sTB via *subclin* | Efficacy lost once individuals progress into sTB | Low = 0.62, 0.74 Medium = 0.74, 0.87  High = 0.87, 1  Zero: 0 | | 50% | 10 years |
| Prevention of any infectious disease | AI excluding disease | Reduces progression to aTB from I and nTB via *nonsub*, *infsub* and *relsub*. | Efficacy lost once individuals progress into aTB | Low = 0.62, 0.74 Medium = 0.74, 0.87  High = 0.87, 1  Zero: 0 | | 50% | 10 years |
| Prevention of any disease | AI excluding disease | Reduces progression from infection to nTB/aTB via *infnon*, *infsub*, *relnon*, and *relsub*. | Efficacy lost once individuals progress into nTB/aTB | Low = 0.62, 0.74 Medium = 0.74, 0.87  High = 0.87, 1  Zero: 0 | | 50% | 10 years |
| Prevention of only infectious symptomatic disease | AI including disease | Reduces progression from aTB to sTB via *subclin* | Efficacy lost once individuals progress into sTB | 0.62, 1 | | 50% | 10 years |
| Prevention of any infectious disease | AI including disease | Reduces progression to aTB from I and nTB via *nonsub*, *infsub* and *relsub*. | Efficacy lost once individuals progress into aTB | 0.62, 1 | | 50% | 10 years |
| Prevention of any disease | AI including disease | Reduces progression from infection to nTB/aTB via *infnon*, *infsub*, *relnon*, and *relsub*. | Efficacy lost once individuals progress into nTB/aTB | 0.62, 1 | | 50% | 10 years |
| Prevention of only infectious symptomatic disease | CI including disease | Reduces progression from aTB to sTB via *subclin* | Efficacy lost once individuals progress into sTB | 0.62, 1 | | 50% | 10 years |
| Prevention of any infectious disease | CI including disease | Reduces progression to aTB from I and nTB via *nonsub*, *infsub* and *relsub*. | Efficacy lost once individuals progress into aTB | 0.62, 1 | | 50% | 10 years |
| Prevention of any disease | CI including disease | Reduces progression from infection to nTB/aTB via *infnon*, *infsub*, *relnon*, and *relsub*. | Efficacy lost once individuals progress into nTB/aTB | 0.62, 1 | | 50% | 10 years |
| **Varying efficacy analysis** | | | | | | | |
| Prevention of only infectious symptomatic disease | AI excluding disease | Reduces progression from aTB to sTB via *subclin* | Efficacy lost once individuals progress into sTB | 0.62, 1 | 25% or 75% | | 10 years |
| Prevention of any infectious disease | AI excluding disease | Reduces progression to aTB from I and nTB via *nonsub*, *infsub* and *relsub*. | Efficacy lost once individuals progress into aTB | 0.62, 1 | 50% | | 10 years |
| Prevention of any disease | AI excluding disease | Reduces progression from infection to nTB/aTB via *infnon*, *infsub*, *relnon*, and *relsub*. | Efficacy lost once individuals progress into nTB/aTB | 0.62, 1 | 25% or 75 | | 10 years |
| **Varying duration of protection analysis** | | | | | | | |
| Prevention of only infectious symptomatic disease | AI excluding disease | Reduces progression from aTB to sTB via *subclin* | Efficacy lost once individuals progress into sTB | 0.62, 1 | 50% | | 5 years or 20 years |
| Prevention of any infectious disease | AI excluding disease | Reduces progression to aTB from I and nTB via *nonsub*, *infsub* and *relsub*. | Efficacy lost once individuals progress into aTB | 0.62, 1 | 50% | | 10 years |
| Prevention of any disease | AI excluding disease | Reduces progression from infection to nTB/aTB via *infnon*, *infsub*, *relnon*, and *relsub*. | Efficacy lost once individuals progress into nTB/aTB | 0.62, 1 | 50% | | 5 years or 20 years |

### *Vaccine model structure*

The vaccine model structure used in this work was described in section 4.3 of the Supplementary Material of Clark et al., *BMC Medicine*, 2023 (1), and reproduced here with modifications.

The vaccine structure for an any infection (AI) vaccine or a current infection (CI) vaccine is in Figure B. Each compartment in the vaccine structure is replicated for all TB natural history compartments and ages. An AI vaccine was assumed to be efficacious with any infection status (aside from current active disease) at the time of vaccination, whereas the CI vaccine was assumed to be efficacious only with current infection at the time of vaccination. We accounted for differences in *Vaccinated Protected*, *Vaccinated Not Protected*, and *Vaccinated Waned*. The *Vaccinated Not Protected* compartments are included as we assume that individuals with may be accidentally vaccinated and would not receive protection from the vaccine. With each vaccine course the level of protection builds if the recipient is currently in a *Vaccinated Protected* compartment. Waning occurs from any of the *Vaccinated Protected* compartments to the *Vaccinated Protected* compartment one level below, or to the *Waned Protection* compartment for those with only one course of protection.


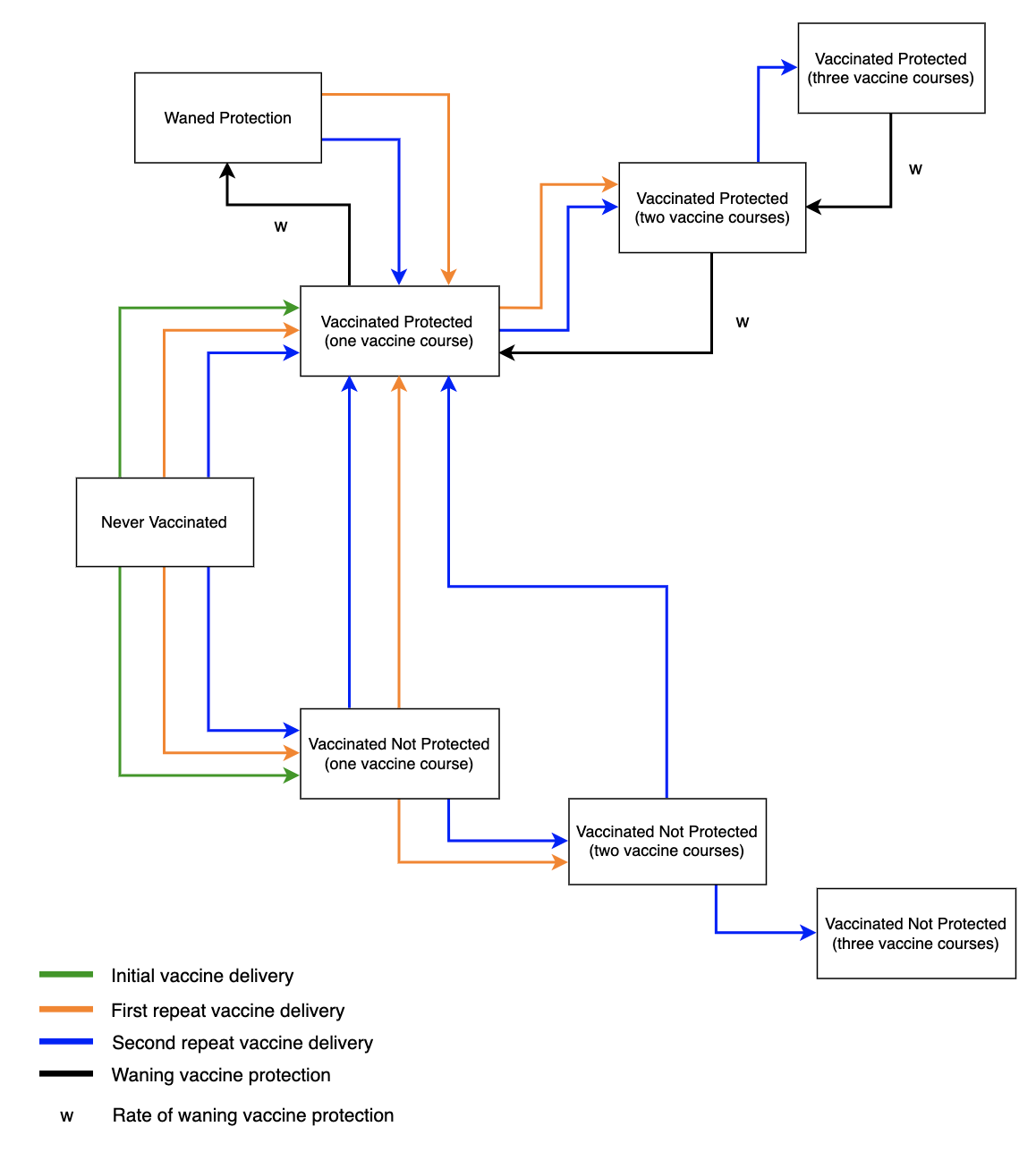


**Figure B Vaccine structure for an AI or CI vaccine**

### *Vaccine integration in the TB natural history model*

Vaccine protection is incorporated in the TB natural history structure as indicated with the dashed arrows in Figure C. The vaccine acts by reducing the rate of progression to disease by $(1-pV)$ depending on vaccine scenario characteristics, where $pV$ is the vaccine efficacy.

For the vaccine preventing progression to infectious symptomatic disease only (the red dashed line), we assumed that $(1-pV)$ would be applied to $subclin$.

For the vaccine preventing progression to any infectious disease (the blue dotted-dashed lines), we assumed that $\left( 1-pV \right)$would be applied to $infsub$, $nonsub$, and $relsub$.

For the vaccine preventing progression to any disease (the grey dotted lines), we assumed that $\left( 1-pV \right)$ would be applied to $infnon$, $infsub$, $relnon$, and $relsub$.

Vaccine efficacy was modelled as “degree”, which assumes that everyone who has been vaccinated and receives protection (those in the *Vaccinated Protected* compartments) will have protection from the vaccine equivalent to the value of the vaccine efficacy.


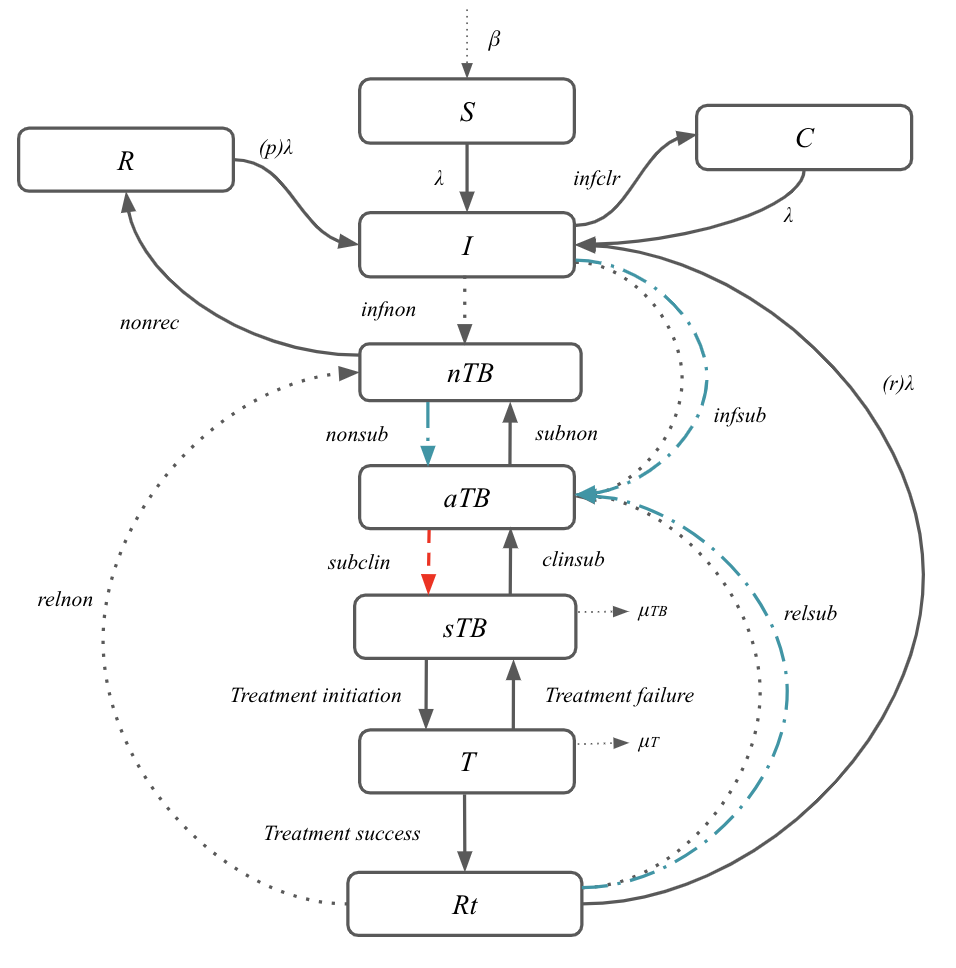


**Figure C** **TB natural history structure indicating where vaccine efficacy is applied**

The host infection status required for vaccine efficacy is indicated with the shaded compartments in Figure D. For scenarios where we assumed that all vaccine mechanisms of action (preventing progression to only infectious symptomatic disease, any infectious disease, or any disease) would not be effective in pre-symptomatic stages, we assumed:

- AI – excluding disease: grey shaded compartments (both solid and lined)
- CI – excluding disease: grey shaded compartments (with lines only)

For scenarios where we assumed the vaccine would be effective in pre-symptomatic stages, we assumed that for the vaccine preventing progression to symptomatic disease only:

- AI – including disease: grey shaded compartments (both solid and lined), the blue compartment, and the red compartment
- CI – including disease: grey shaded compartments (with lines only), the blue compartment, and the red compartment

For the vaccine preventing progression to any infectious disease:

- AI – including disease: grey shaded compartments (both solid and lined) and the blue compartment
- CI – including disease: grey shaded compartments (with lines only) and the blue compartment

There was no scenario including disease for a vaccine preventing progression to any disease, as there were no pre-symptomatic stages before where the vaccine would exert the prevention of disease effect.

**
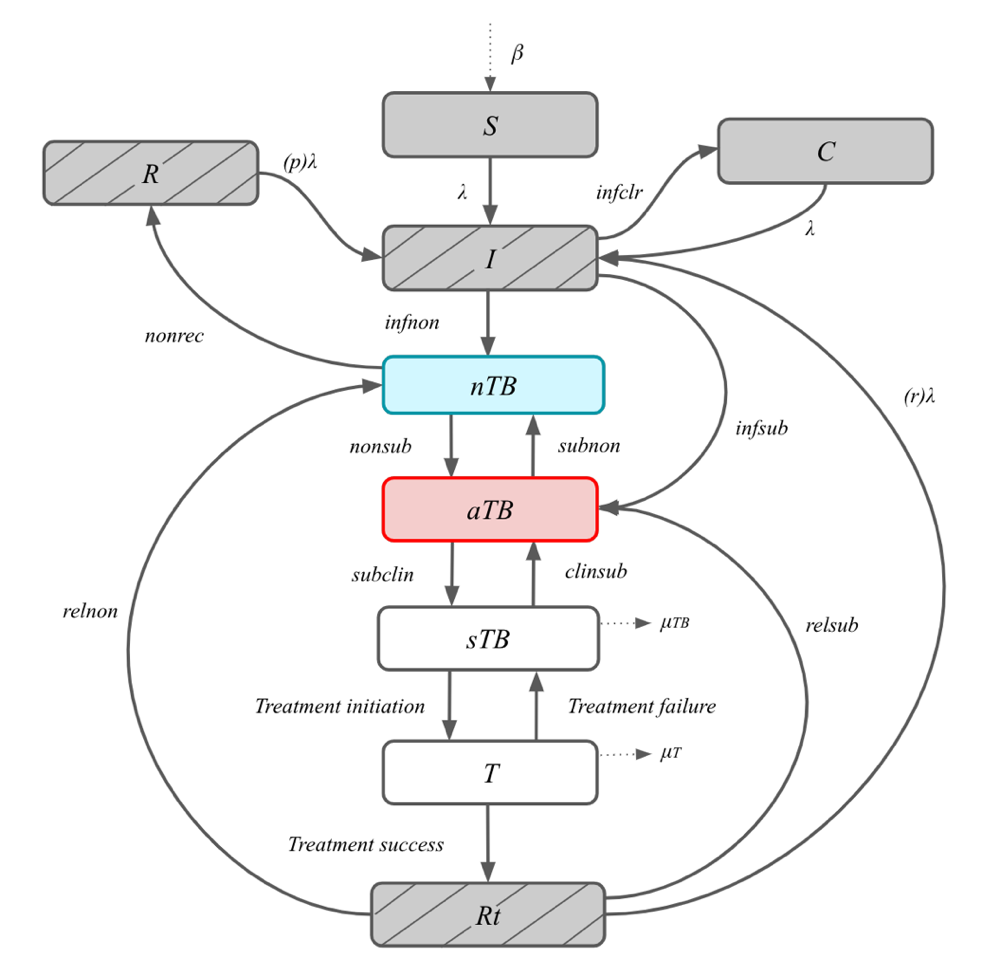
**

**Figure D** **TB natural history structure indicating host infection status required for efficacy**

# SUPPORTING RESULTS

## No-new-vaccine scenario results

### 5.1 No-new-vaccine calibration (with baseline, low, medium and high relative infectiousness)

Figure E shows trends in TB incidence, TB case notifications, infectious TB disease prevalence, TB mortality, proportion of infectious TB that is asymptomatic, and TB infection prevalence from 2005–2050 for all ages, based on 500 parameter sets calibrated to all 14 targets under varying assumptions about relative asymptomatic TB infectiousness: baseline (0.62, 1), low (0.62, 0.74), medium (0.74, 0.87), and high (0.87, 1).

***
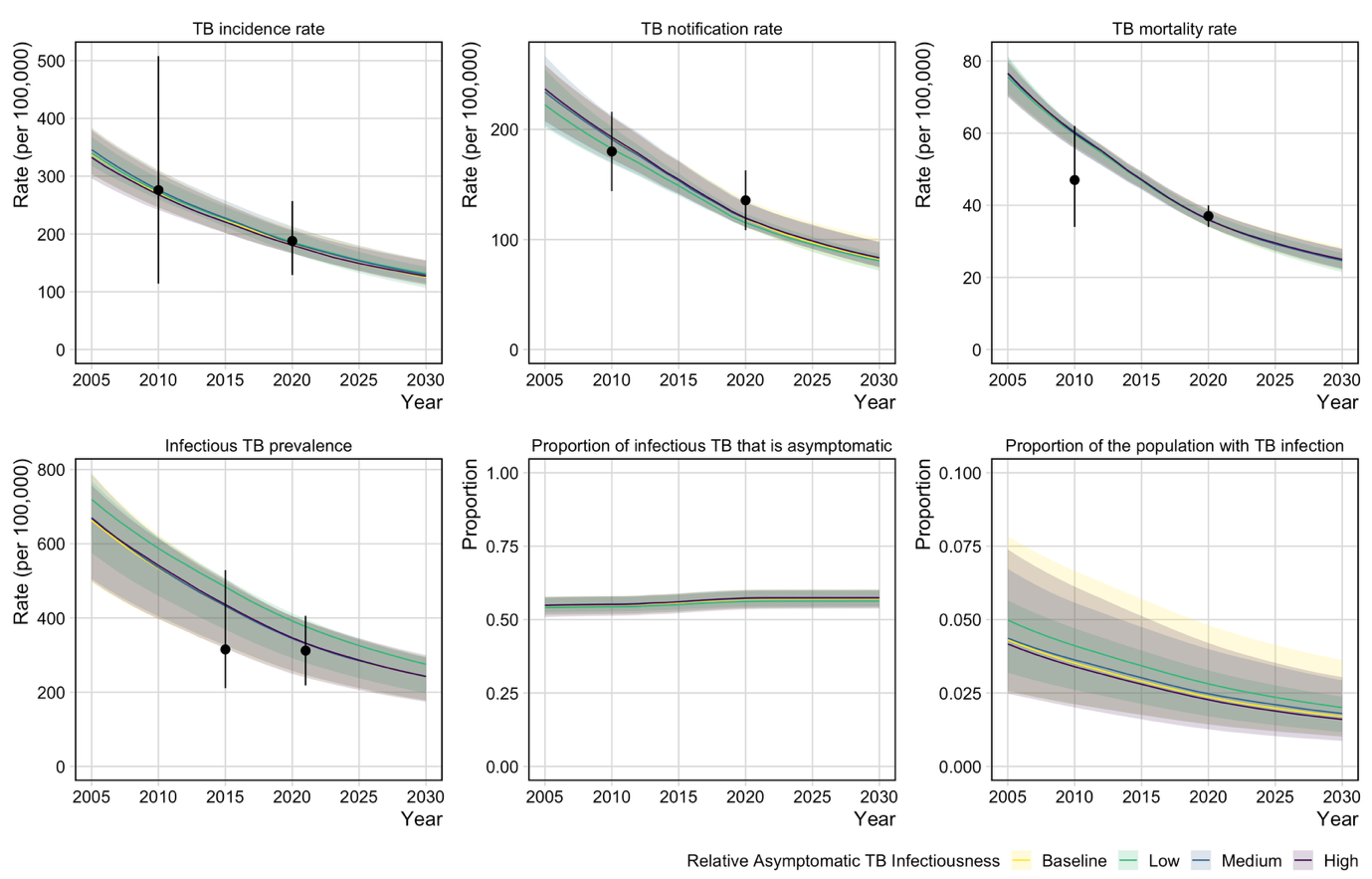
***

Figure E Trends in TB epidemiology from 2005–2050 for all ages for model calibrations under varying assumptions about relative asymptomatic TB infectiousness: baseline (0.62, 1), low (0.62, 0.74), medium (0.74, 0.87), and high (0.87, 1). The trend lines in yellow, green, blue and purple indicate the median modelled output with 95% uncertainty reflected by the respective shaded colours. The black dot and vertical lines are the calibration targets from Table E. Note y-axis scales differ.

### 5.2 No-new-vaccine scenario calibration (with zero relative infectiousness)

Figure F shows trends in TB incidence, TB case notifications, infectious TB disease prevalence, TB mortality, proportion of infectious TB that is asymptomatic, and TB infection prevalence from 2005–2050 for all ages, based on 500 parameter sets calibrated to 14 out of 14 calibration targets under zero relative aTB infectiousness (Figure F).


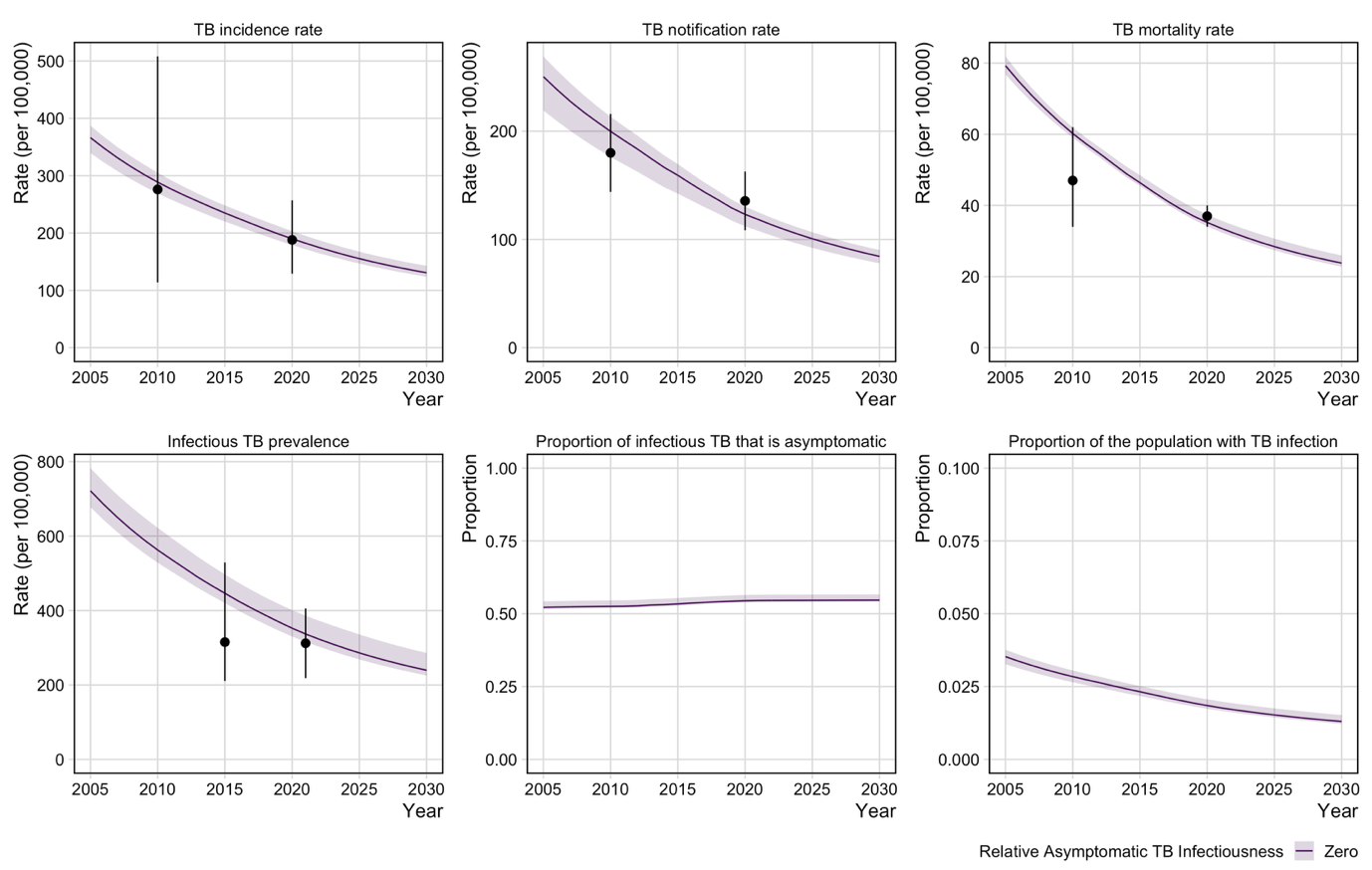


Figure F Trends in TB epidemiology from 2005–2050 for all ages for zero relative aTB infectiousness. The black dot and vertical lines are the calibration targets from Table E. Note y-axis scales differ.

Table G shows the number of sTB, aTB, and nTB episodes, the overall number of infectious episodes (aTB + sTB), and TB deaths for the no-new-vaccine scenario under the baseline and zero aTB relative infectiousness scenarios.

Table G No-new-vaccine baseline incidence of TB episodes and deaths (millions), under baseline and zero aTB infectiousness relative to sTB

| Numbers, millions (95% UI) | Short-term (2030–2032) | | Longer-term (2030–2050) | | |  |
| --- | --- | --- | --- | --- | --- | --- |
|  | **Baseline infectiousness (0.62, 1)** | **Zero infectiousness  (0)** | | **Baseline infectiousness (0.62, 1)** | **Zero infectiousness  (0)** | |
| sTB | 6.5 (5.7, 7.9) | 6.8  (6.3, 7.3) | | 38.1  (32.1, 48.4) | 40.1  (36.7, 44.9) | |
| aTB | 16.3  (12.9, 20.4) | 17.1  (15.7, 20.2) | | 95.4  (72.7, 123.2) | 101.9  (92.7, 123.8) | |
| aTB + sTB | 22.8  (18.6, 27.8) | 23.9  (22, 27.5) | | 133.8  (104.6, 169.8) | 141.8  (129.7, 168.4) | |
| nTB | 19.6  (12.1, 27.8) | 23.8  (21.7, 28.4) | | 115.0  (67.1, 166.1) | 142.3  (128.8, 175.6) | |
| TB deaths | 1.1  (1.0, 1.3) | 1.1  (1.0, 1.2) | | 6.4  (5.4, 7.7) | 6.2  (5.9, 7.3) | |

*Abbreviations: aTB = infectious asymptomatic TB; nTB = non-*infectious *TB; sTB = infectious symptomatic TB*

Figure G shows the posterior distribution of varying parameters in the baseline relative infectiousness scenario plotted on their prior ranges from Table A.


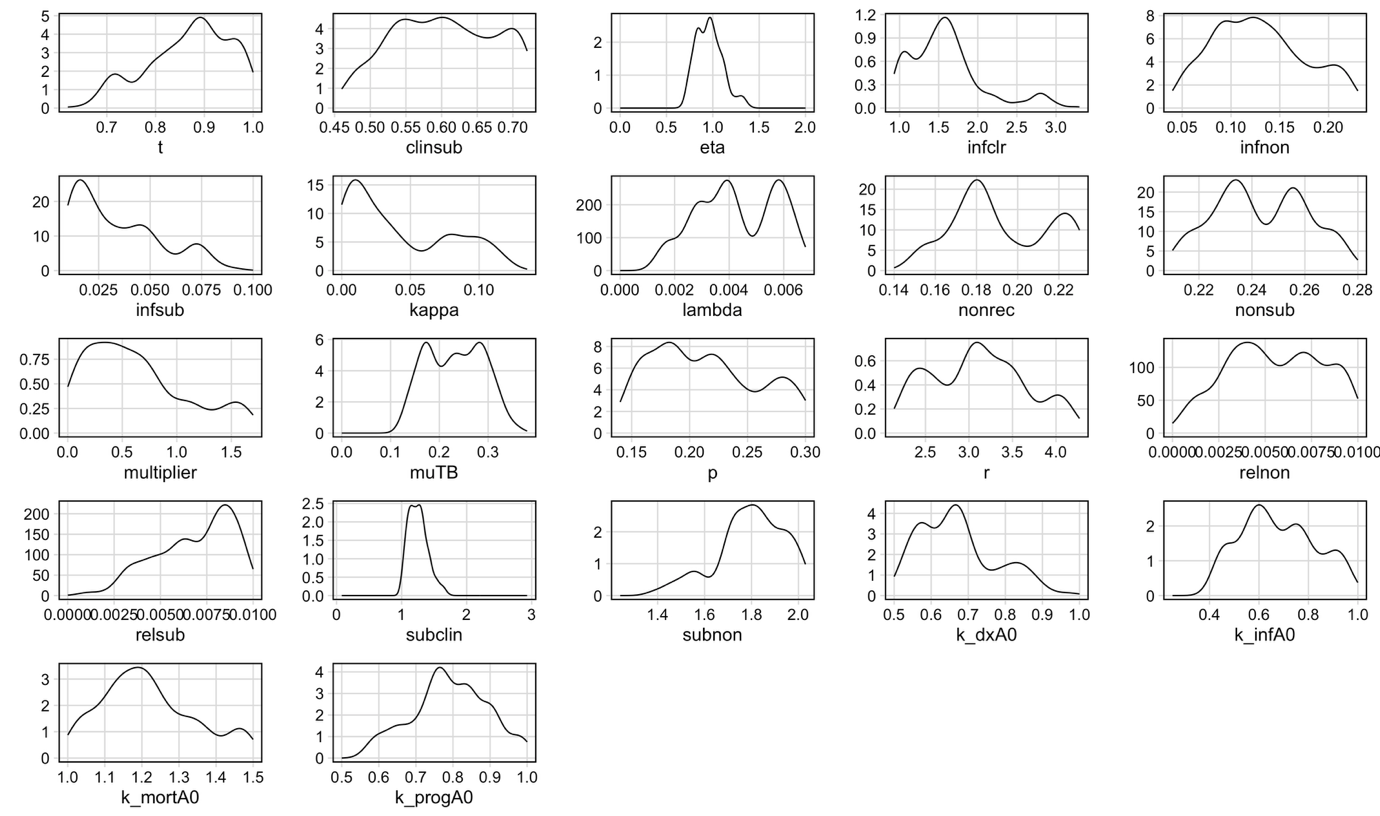


Figure G Posterior distribution of varying parameters in the baseline relative infectiousness scenario

## Vaccinated proportions over time

Figure H shows the annual vaccinated proportions between 2030 and 2050 for ages 15, 25 and 40-year-olds across the *Basecase* scenarios for a randomly selected parameter set.


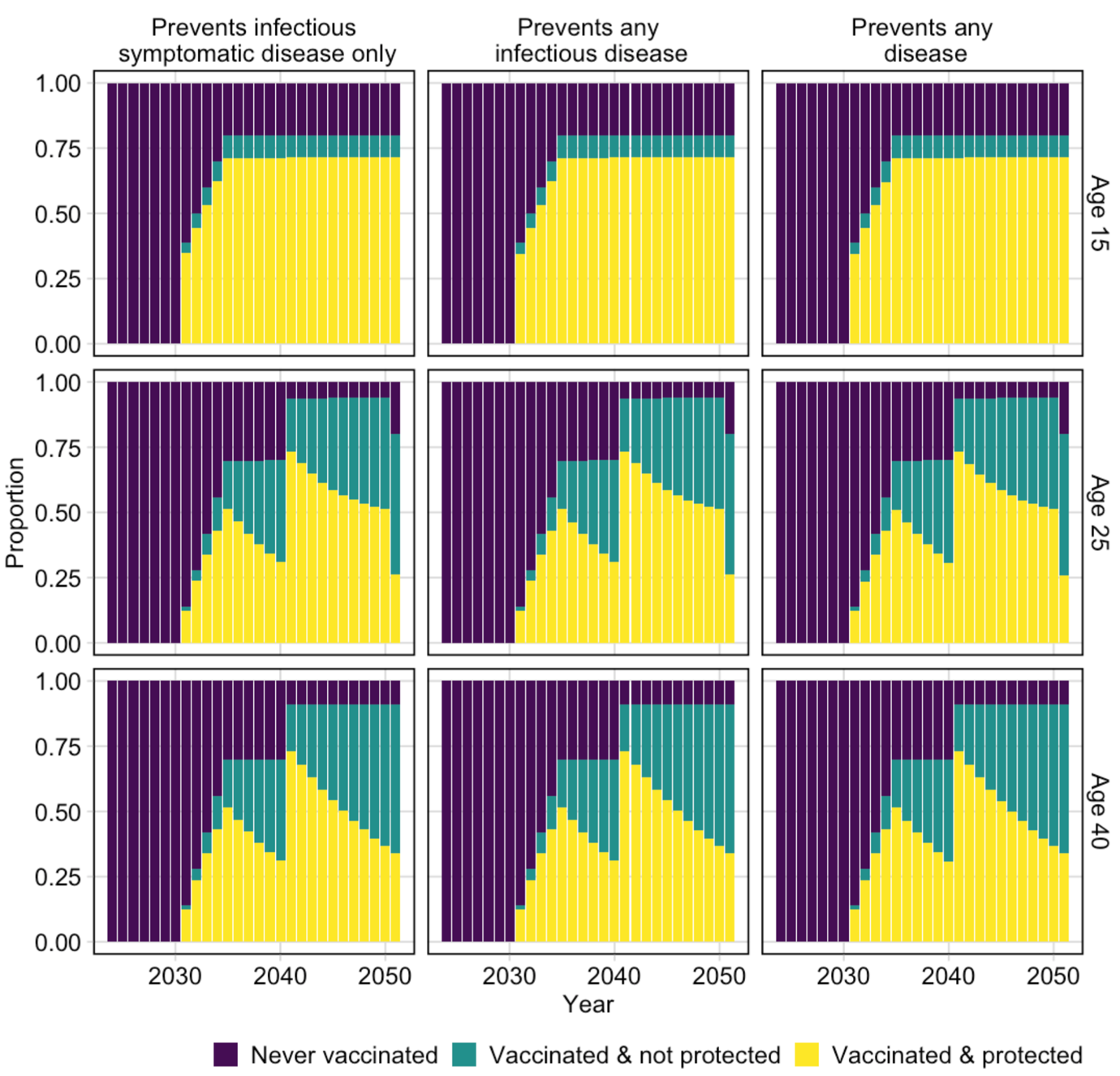


Figure H Proportion of population vaccinated from 2030–2050, for ages 15, 25 and 40 years old.

## Proportion in each TB state over time

Figure I shows the proportion in each TB state between 2025 and 2050 across the No-new-vaccine baseline and *Basecase* scenarios for a randomly selected parameter set.

**
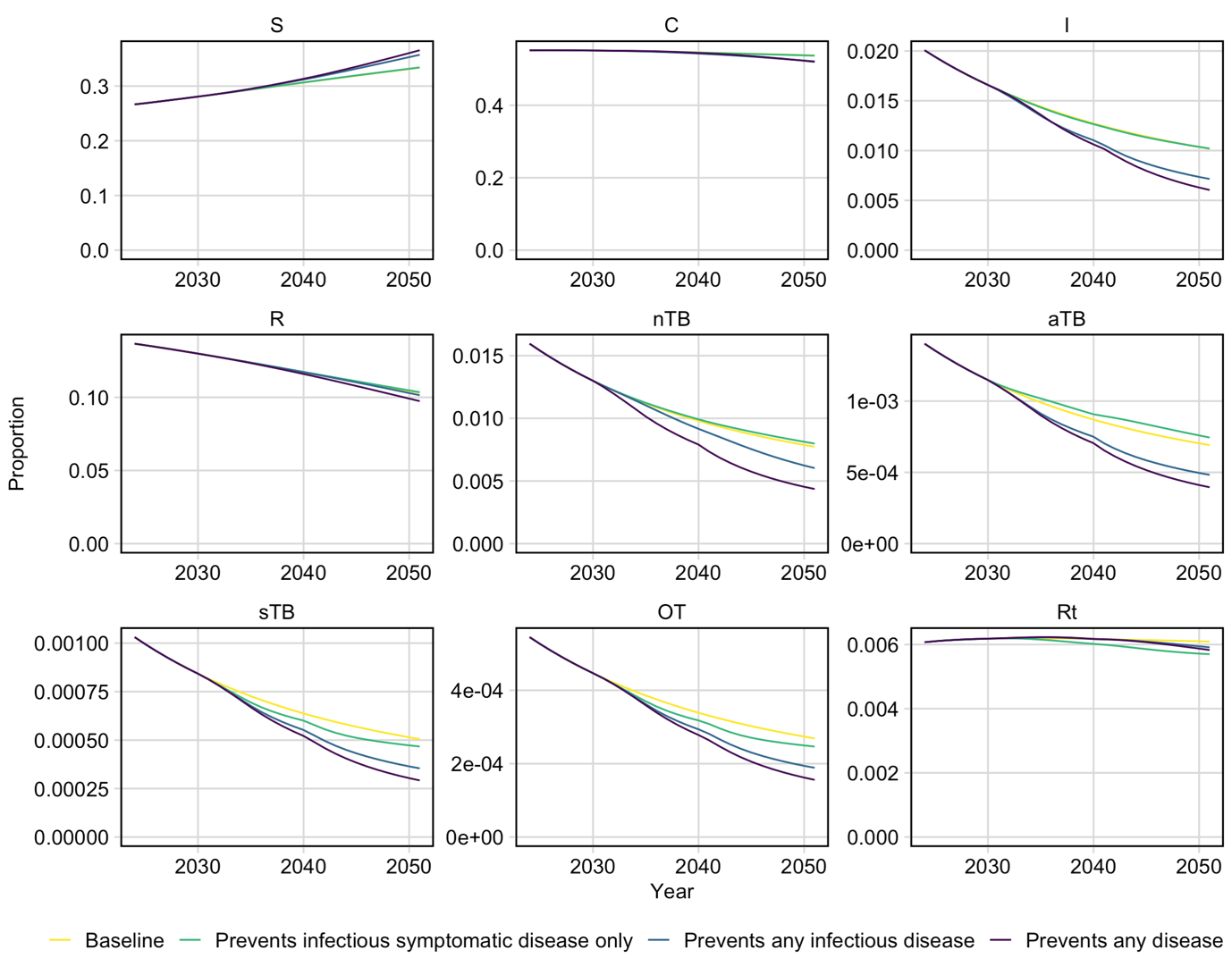
**

Figure I Proportion in each TB state between 2025 and 2050 across the No-new-vaccine baseline and Basecase scenarios for a randomly selected parameter set. Note y-axis scales differ. *Abbreviations: aTB = infectious asymptomatic TB; nTB = non-infectious TB; sTB = infectious symptomatic TB*

*
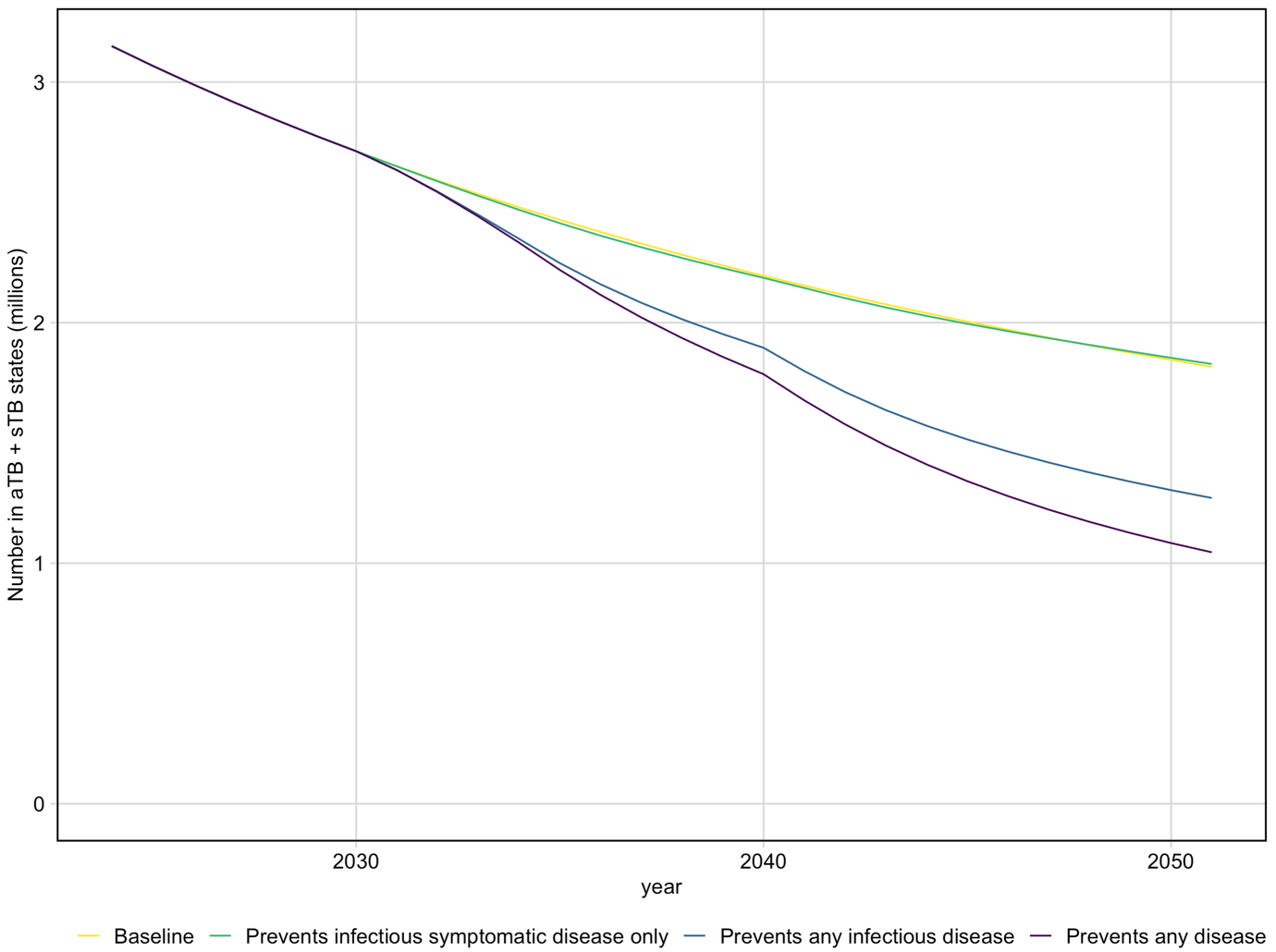
*

Figure J Prevalence of aTB and sTB between 2025 and 2050 across the No-new-vaccine baseline and Basecase scenarios for a randomly selected parameter set, weighted by the relative infectiousness of aTB. *Abbreviations: aTB = infectious asymptomatic TB; sTB = infectious symptomatic TB.*

## Sensitivity analysis results: vaccines effective with current infection status

Figure K and Table H shows the short-term and longer-term impact of vaccines that are effective in current infection status at the time of vaccination. Results showed that vaccines effective only in infected individuals had lower impact at all time points compared to the *Basecase* analysis but displayed similar patterns and trends in their mechanism of action to vaccines effective in both uninfected and infected individuals.


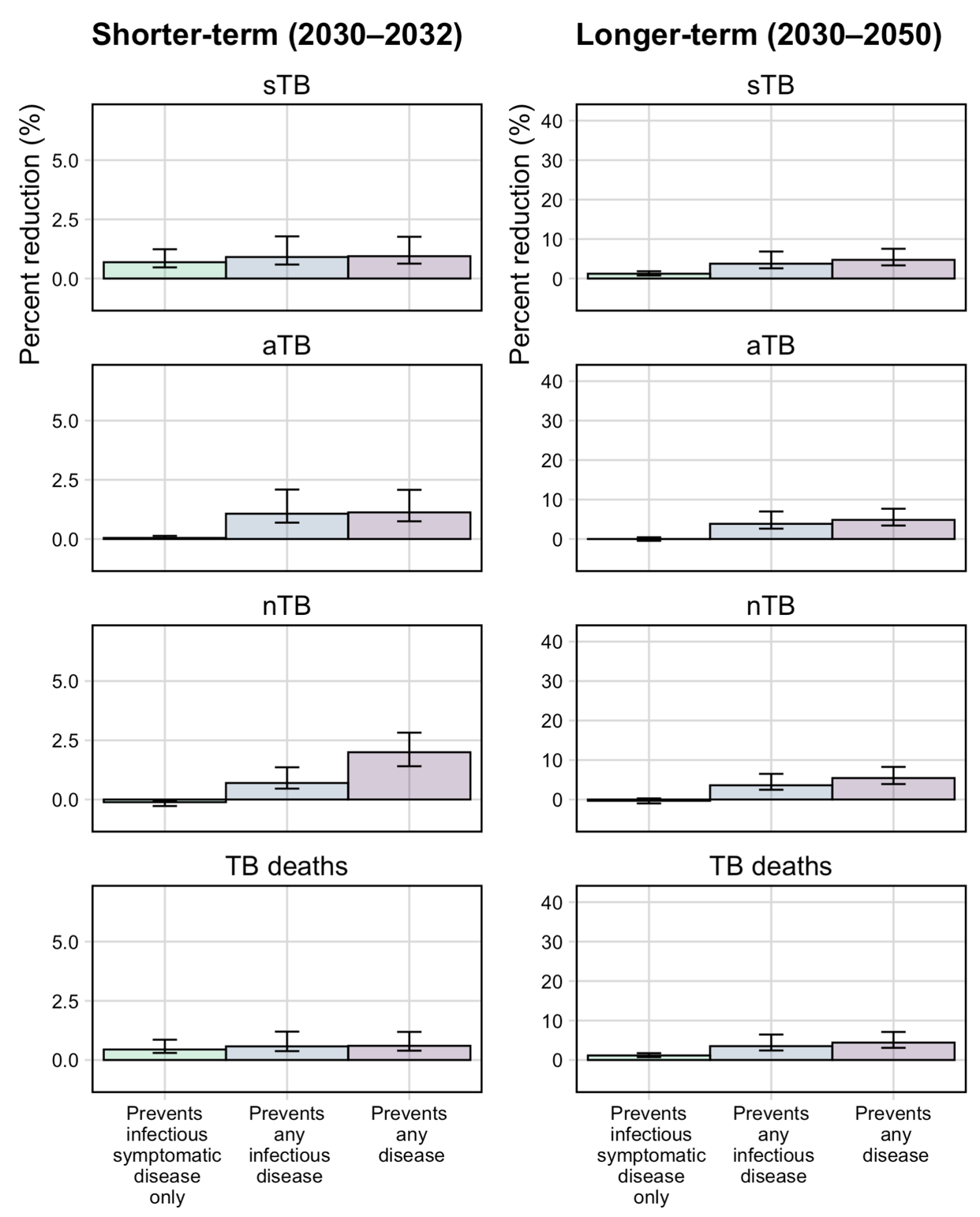


Figure K Percentage cumulative TB episodes and deaths averted between 2030–2032 (left column) or between 2030–2050 (right column) for a current infection vaccine. Error bars represent 95% uncertainty intervals. Note y-axis scales differ. *Abbreviations: aTB = infectious asymptomatic TB; nTB = non-infectious TB; sTB = infectious symptomatic TB.*

Table H Number and percentage cumulative TB episodes and deaths averted (2030–2032 and 2030–2050) for vaccines effective with current infection under baseline aTB infectiousness

|  | | **2030–2032** | | | **2030–2050** | | |
| --- | --- | --- | --- | --- | --- | --- | --- |
| **Vaccine prevents progression to:** | | **Infectious symptomatic disease only** | **Any infectious disease** | **Any**  **disease** | **Infectious symptomatic disease only** | **Any infectious disease** | **Any**  **disease** |
| **Episodes averted (millions)** | sTB | 0.05  (0.03, 0.09) | 0.06  (0.04, 0.14) | 0.06  (0.04, 0.13) | 0.47  (0.27, 0.85) | 1.38  (0.90, 3.17) | 1.79  (1.20, 3.44) |
|  | aTB | 0.01  (0.00, 0.02) | 0.17  (0.11, 0.37) | 0.18  (0.11, 0.37) | -0.01  (-0.41, 0.52) | 3.56  (2.27, 7.51) | 4.68  (3.09, 8.28) |
|  | aTB + sTB | 0.05  (0.03, 0.12) | 0.23  (0.14, 0.51) | 0.24  (0.15, 0.50) | 0.42  (0.01, 1.23) | 4.93  (3.21, 10.70) | 6.49  (4.34, 11.76) |
|  | nTB | -0.02  (-0.05, -0.01) | 0.14  (0.09, 0.24) | 0.40  (0.24, 0.59) | -0.40  (-1.07, 0.42) | 4.21  (2.47, 7.20) | 6.64  (3.67, 10.18) |
|  | TB deaths | 0.00  (0.00, 0.01) | 0.01  (0.00, 0.01) | 0.01  (0.00, 0.01) | 0.08  (0.04, 0.12) | 0.21  (0.14, 0.45) | 0.27  (0.19, 0.51) |
| **Percent reduction (%)** | sTB | 0.69  (0.47, 1.23) | 0.91  (0.59, 1.78) | 0.94  (0.63, 1.76) | 1.21  (0.76, 1.83) | 3.76  (2.57, 6.83) | 4.72  (3.33, 7.53) |
|  | aTB | 0.05  (0.03, 0.13) | 1.07  (0.69, 2.09) | 1.12  (0.75, 2.08) | -0.01  (-0.47, 0.45) | 3.85  (2.63, 6.98) | 4.85  (3.42, 7.68) |
|  | aTB + sTB | 0.24  (0.16, 0.46) | 1.02  (0.66, 2.00) | 1.07  (0.71, 1.98) | 0.32  (0.01, 0.79) | 3.83  (2.61, 6.94) | 4.82  (3.39, 7.63) |
|  | nTB | -0.11  (-0.28, -0.04) | 0.70  (0.46, 1.36) | 2.00  (1.41, 2.82) | -0.33  (-0.99, 0.27) | 3.61  (2.47, 6.49) | 5.43  (3.91, 8.25) |
|  | TB deaths | 0.44  (0.30, 0.86) | 0.58  (0.37, 1.20) | 0.60  (0.39, 1.19) | 1.15  (0.72, 1.74) | 3.51  (2.40, 6.45) | 4.41  (3.08, 7.10) |

*Abbreviations: aTB = infectious asymptomatic TB; nTB = non-infectious TB; sTB = infectious symptomatic TB*

## Trends over time for vaccines effective with current infection status

Figure L shows the trends in the number of sTB, aTB, and nTB episodes and TB deaths between 2030–2050 for each current infection vaccine scenario compared to no-new-vaccine scenario, under baseline relative aTB infectiousness (0.62, 1).


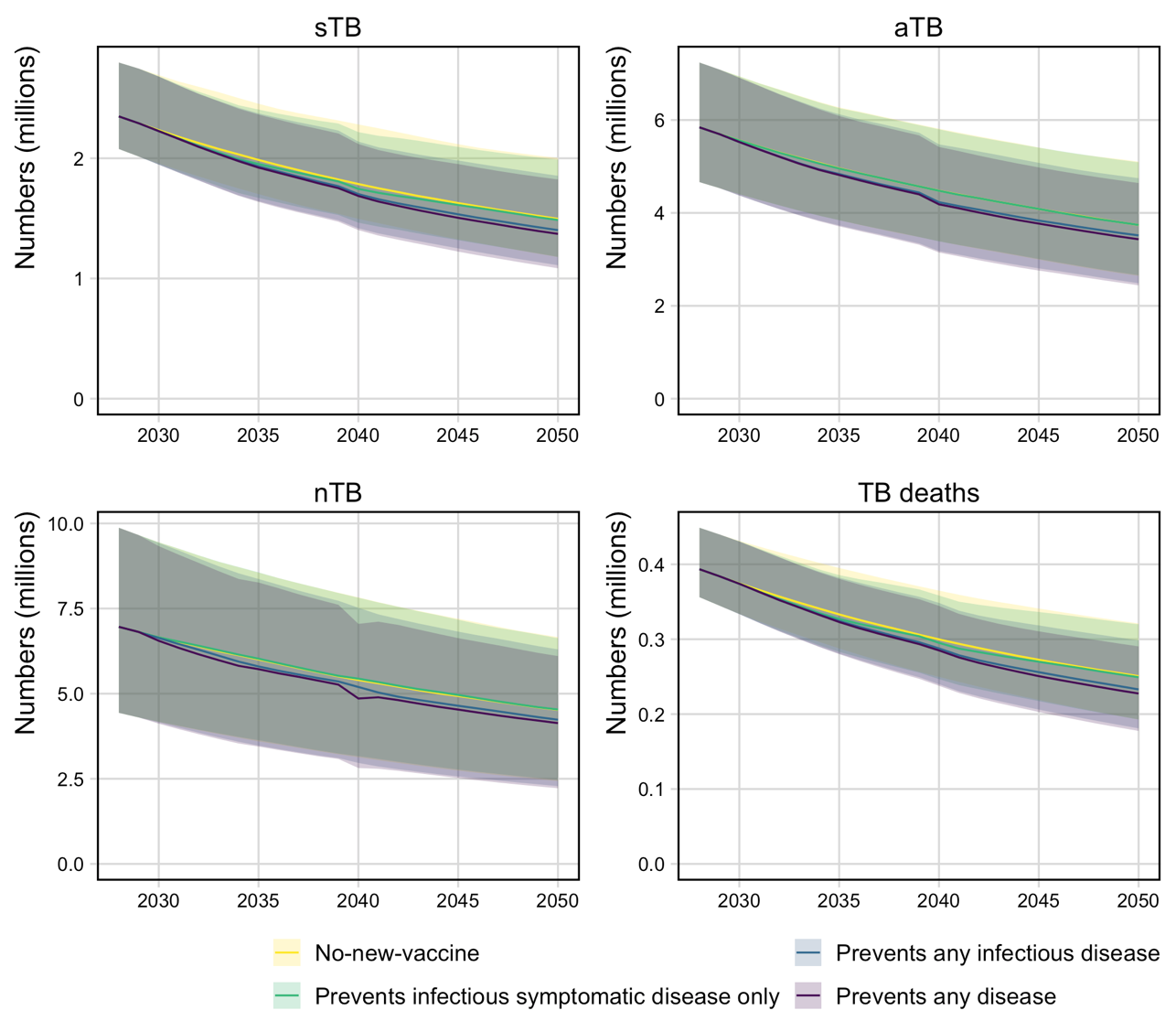


Figure L Trends in the number of sTB, aTB, and nTB episodes and TB deaths between 2030–2050 for each current infection vaccine scenario compared to no-new-vaccine scenario for baseline aTB infectiousness. Shaded areas represent 95% uncertainty intervals. Note y-axis scales differ. *Abbreviations: aTB = infectious asymptomatic TB; nTB = non-infectious TB; sTB = infectious symptomatic TB.*

## Sensitivity analysis results: vaccines effective with any infection status with varying infectiousness

### 9.1 Short-term impact (2030–2032)

The short-term impact of vaccines that are effective in any infection status at the time of vaccination under varying assumptions about the relative infectiousness of aTB is in Figure M and Table I. Infectiousness scenarios were based on current literature estimates (0.62, 1) and divided into low (0.62, 0.74), medium (0.74, 0.87), and high (0.87, 1) ranges, with an additional scenario assuming zero infectiousness. Results showed that the short-term impact of vaccines across all infectiousness scenarios was similar to the *Basecase* vaccine scenario impact estimates, which assumed baseline infectiousness (0.62, 1) (Figure M, Table I).


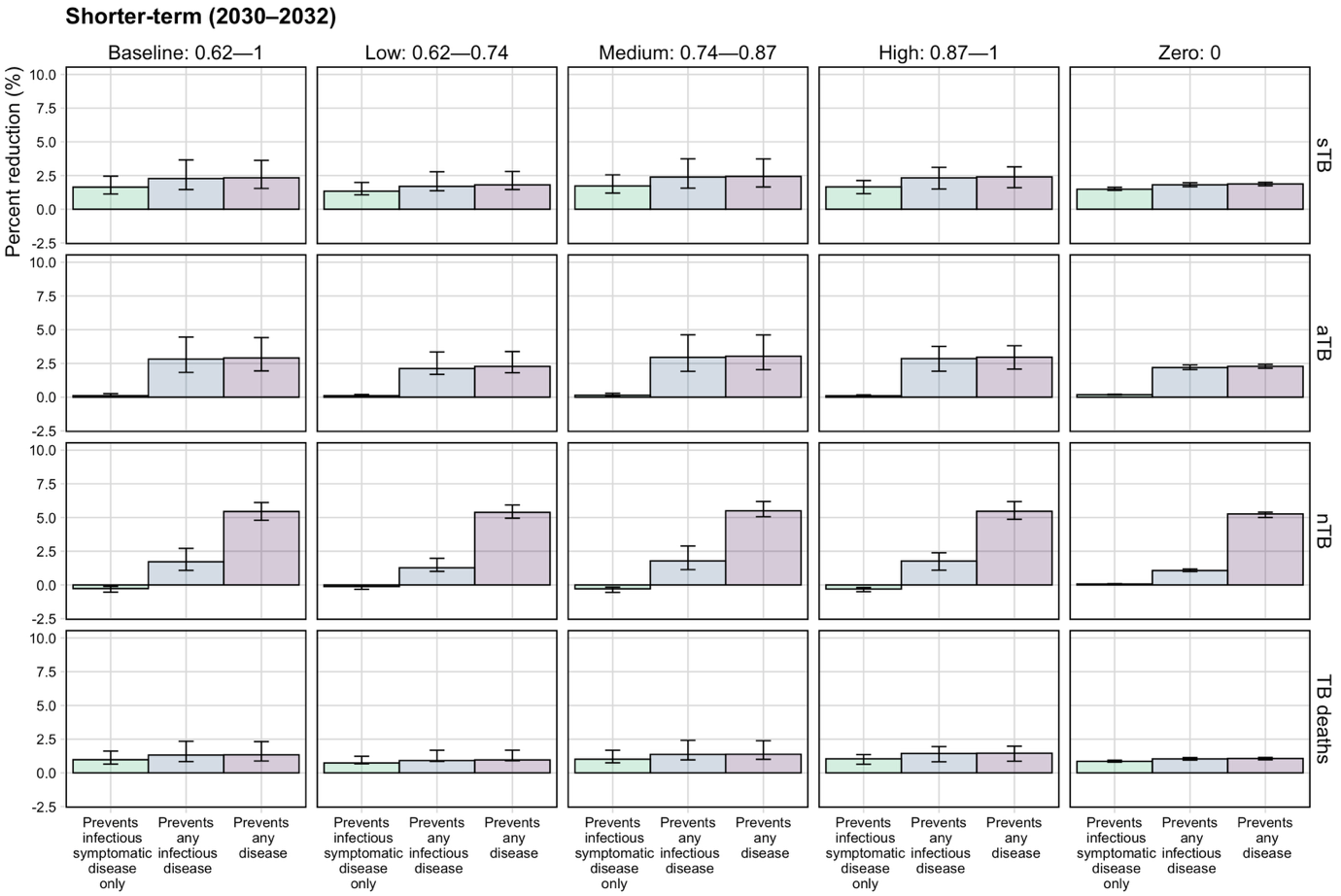


Figure M Percentage cumulative TB episodes and deaths averted between 2030–2032 for an any infection vaccine by varied relative infectiousness of aTB. Error bars represent 95% uncertainty intervals.
*Abbreviations: aTB = infectious asymptomatic TB; nTB = Non-infectious TB; sTB = infectious symptomatic TB*

Table I Number and percentage cumulative TB episodes and deaths averted between 2030–2032 for vaccines effective in any infection, under low, medium, high and zero asymptomatic TB infectiousness relative to symptomatic TB

|  |  | **Episodes Averted (millions)** | | | **Percent reduction (%)** | | |
| --- | --- | --- | --- | --- | --- | --- | --- |
| **Vaccine prevents progression to:** | | **Infectious symptomatic disease only** | **Any infectious disease** | **Any**  **disease** | **Infectious symptomatic disease only** | **Any infectious disease** | **Any**  **disease** |
| **Low infectiousness**  **(0.62, 0.74)** | sTB | 0.09  (0.07, 0.13) | 0.12  (0.08, 0.19) | 0.12  (0.09, 0.19) | 1.3  (1.1, 2.0) | 1.7  (1.4, 2.8) | 1.8  (1.5, 2.8) |
|  | aTB | 0.02  (0.01, 0.03) | 0.40  (0.26, 0.55) | 0.43  (0.27, 0.56) | 0.1  (0.1, 0.2) | 2.1  (1.7, 3.4) | 2.3  (1.8, 3.4) |
|  | aTB + sTB | 0.11  (0.08, 0.17) | 0.52  (0.34, 0.73) | 0.55  (0.36, 0.74) | 0.4  (0.4, 0.7) | 2.0  (1.6, 3.2) | 2.1  (1.7, 3.2) |
|  | nTB | -0.03  (-0.06, -0.03) | 0.33  (0.21, 0.40) | 1.38  (0.85, 1.54) | -0.1  (-0.3, -0.1) | 1.3  (1.0, 2.0) | 5.4  (4.9, 5.9) |
|  | TB deaths | 0.01  (0.01, 0.01) | 0.01  (0.01, 0.02) | 0.01  (0.01, 0.02) | 0.7  (0.7, 1.2) | 0.9  (0.8, 1.7) | 1.0  (0.9, 1.7) |
| **Medium infectiousness**  **(0.74, 0.87)** | sTB | 0.11  (0.08, 0.20) | 0.15  (0.11, 0.30) | 0.16  (0.11, 0.29) | 1.7  (1.2, 2.6) | 2.4  (1.6, 3.7) | 2.4  (1.7, 3.7) |
|  | aTB | 0.02  (0.01, 0.05) | 0.48  (0.32, 0.86) | 0.49  (0.34, 0.85) | 0.1  (0.1, 0.3) | 3.0  (1.9, 4.6) | 3.0  (2.0, 4.6) |
|  | aTB + sTB | 0.13  (0.09, 0.25) | 0.63  (0.42, 1.16) | 0.64  (0.45, 1.14) | 0.6  (0.4, 1.0) | 2.8  (1.8, 4.3) | 2.9  (1.9, 4.3) |
|  | nTB | -0.05  (-0.1, -0.04) | 0.35  (0.23, 0.53) | 1.1  (0.78, 1.46) | -0.3  (-0.6, -0.2) | 1.8  (1.1, 2.9) | 5.5  (5.1, 6.2) |
|  | TB deaths | 0.01  (0.01, 0.02) | 0.01  (0.01, 0.03) | 0.01  (0.01, 0.03) | 1.0  (0.7, 1.7) | 1.4  (1.0, 2.4) | 1.4  (1.0, 2.4) |
| **High infectiousness**  **(0.87, 1)** | sTB | 0.11  (0.07, 0.15) | 0.15  (0.1, 0.22) | 0.16  (0.10, 0.22) | 1.7  (1.2, 2.1) | 2.3  (1.5, 3.1) | 2.4  (1.6, 3.1) |
|  | aTB | 0.02  (0.01, 0.03) | 0.46  (0.28, 0.68) | 0.47  (0.3, 0.69) | 0.1  (0.1, 0.2) | 2.9  (1.9, 3.8) | 3.0  (2.1, 3.8) |
|  | aTB + sTB | 0.12  (0.09, 0.18) | 0.62  (0.38, 0.89) | 0.63  (0.41, 0.90) | 0.6  (0.4, 0.7) | 2.7  (1.8, 3.6) | 2.8  (1.9, 3.6) |
|  | nTB | -0.06  (-0.08, -0.03) | 0.32  (0.19, 0.49) | 1.01  (0.64, 1.46) | -0.3  (-0.5, -0.2) | 1.8  (1.1, 2.4) | 5.5  (4.9, 6.2) |
|  | TB deaths | 0.01  (0.01, 0.02) | 0.02  (0.01, 0.02) | 0.02  (0.01, 0.02) | 1.0  (0.6, 1.4) | 1.4  (0.8, 1.9) | 1.5  (0.9, 2.0) |
| **Zero infectiousness**  **(0)** | sTB | 0.10  (0.09, 0.12) | 0.12  (0.11, 0.14) | 0.13  (0.12, 0.14) | 1.5  (1.4, 1.6) | 1.8  (1.7, 2.0) | 1.9  (1.7, 2.0) |
|  | aTB | 0.03  (0.03, 0.04) | 0.38  (0.34, 0.46) | 0.39  (0.36, 0.48) | 0.2  (0.2, 0.2) | 2.2  (2.0, 2.4) | 2.3  (2.1, 2.4) |
|  | aTB + sTB | 0.13  (0.12, 0.16) | 0.50  (0.45, 0.60) | 0.52  (0.47, 0.62) | 0.5  (0.5, 0.6) | 2.1  (1.9, 2.3) | 2.2  (2.0, 2.3) |
|  | nTB | 0.02  (0.01, 0.03) | 0.25  (0.23, 0.32) | 1.25  (1.13, 1.47) | 0.1  (0.1, 0.1) | 1.1  (1.0, 1.2) | 5.3  (5.0, 5.4) |
|  | TB deaths | 0.01  (0.01, 0.01) | 0.01  (0.01, 0.01) | 0.01  (0.01, 0.01) | 0.9  (0.8, 0.9) | 1.0  (0.9, 1.1) | 1.1  (1.0, 1.1) |

*Abbreviations: aTB = infectious asymptomatic TB; nTB = non-infectious TB; sTB = infectious symptomatic TB*.

### 9.2 Longer-term impact (2030–2032)

Figure N and Table J show the longer-term impact of vaccines that are effective with any infection status at the time of vaccination under varying assumptions about the relative infectiousness of aTB. Results showed that the longer-term impact of vaccines under the low (0.62, 0.74), medium (0.74, 0.87), and high (0.87, 1) infectiousness scenarios was similar to the *Basecase* vaccine scenario impact estimates, which assumed baseline infectiousness (0.62–1). However, under the zero infectiousness scenario (assuming aTB is not infectious), vaccines that prevented progression to any infectious disease or any disease showed reduced impact across all measured outcomes, whereas the vaccine that prevented progression to only infectious symptomatic disease showed increased impact across all measured outcomes compared to the *Basecase* vaccine scenario impact estimates (Figure N, Table J).


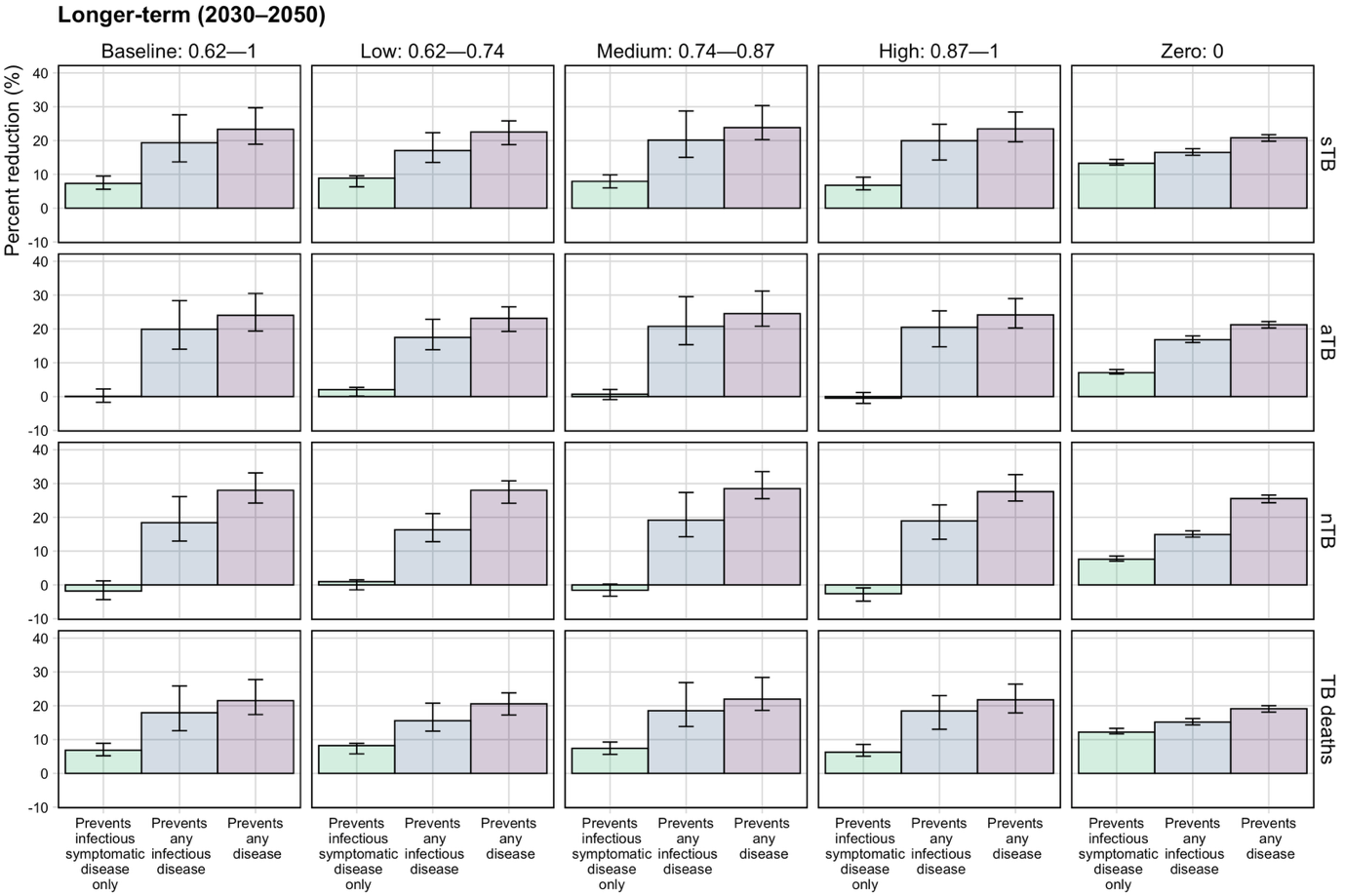


Figure N Percentage cumulative TB episodes and deaths averted between 2030-2050 for an any infection vaccine by varied relative infectiousness of aTB. Error bars represent 95% uncertainty intervals.

*Abbreviations: aTB = infectious asymptomatic TB; nTB = non-infectious TB; sTB = infectious symptomatic TB*

Table J Number and percentage cumulative TB episodes and deaths averted between 2030–2050 for vaccines effective in any infection, under low, medium, high and zero asymptomatic TB infectiousness relative to symptomatic TB.

|  |  | **Numbers averted (millions)** | | | **Percent reduction (%)** | | |
| --- | --- | --- | --- | --- | --- | --- | --- |
| **Vaccine prevents progression to:** | | **Infectious symptomatic disease only** | **Any infectious disease** | **Any**  **disease** | **Infectious symptomatic disease only** | **Any infectious disease** | **Any**  **disease** |
| **Low infectiousness**  **(0.62, 0.74)** | sTB | 3.53  (2.15, 4.06) | 6.85  (4.61, 8.83) | 9.00  (6.40, 10.63) | 8.9  (6.3, 9.5) | 17.0  (13.5, 22.3) | 22.5  (18.8, 25.8) |
|  | aTB | 2.25  (0.12, 3.00) | 19.09  (11.82, 23.31) | 25.11  (16.32, 28.61) | 2.1  (0.1, 2.7) | 17.5  (13.9, 22.9) | 23.2  (19.3, 26.5) |
|  | aTB + sTB | 5.78  (2.27, 6.96) | 26.00  (16.43, 32.07) | 34.29  (22.72, 38.80) | 3.9  (1.9, 4.6) | 17.4  (13.8, 22.7) | 23.0  (19.1, 26.3) |
|  | nTB | 1.46  (-1.40, 2.30) | 24.45  (14.60, 28.64) | 41.57  (26.58, 47.88) | 1.0  (-1.4, 1.5) | 16.3  (12.8, 21.1) | 28  (24.2, 30.8) |
|  | TB deaths | 0.51  (0.34, 0.60) | 0.98  (0.74, 1.30) | 1.28  (1.02, 1.54) | 8.2  (5.8, 8.9) | 15.6  (12.5, 20.7) | 20.6  (17.3, 23.8) |
| **Medium infectiousness**  **(0.74, 0.87)** | sTB | 3.04  (2.16, 4.84) | 7.66  (5.77, 14.11) | 9.32  (7.39, 15.12) | 7.9  (6.0, 9.9) | 20.1  (15, 28.7) | 23.8  (20.3, 30.3) |
|  | aTB | 0.65  (-0.65, 2.3) | 19.61  (13.95, 34.47) | 23.95  (17.54, 37.15) | 0.7  (-0.9, 2.1) | 20.8  (15.4, 29.5) | 24.5  (20.8, 31.2) |
|  | aTB + sTB | 3.65  (1.5, 6.69) | 27.45  (19.82, 48.49) | 33.2  (25.09, 51.95) | 2.8  (1.3, 4.3) | 20.6  (15.3, 29.3) | 24.3  (20.6, 31.0) |
|  | nTB | -1.86  (-3.12, 0.34) | 21.71  (14.82, 32.55) | 34.31  (22.94, 45.65) | -1.6  (-3.3, 0.2) | 19.2  (14.3, 27.3) | 28.5  (25.5, 33.5) |
|  | TB deaths | 0.47  (0.33, 0.69) | 1.16  (0.88, 2.01) | 1.42  (1.12, 2.14) | 7.4  (5.6, 9.2) | 18.5  (13.9, 26.8) | 22.0  (18.6, 28.4) |
| **High infectiousness**  **(0.87, 1)** | sTB | 2.62  (1.84, 4.02) | 7.39  (5.01, 10.44) | 8.92  (6.92, 12.49) | 6.8  (5.4, 9.2) | 20.0  (14.3, 24.8) | 23.5  (19.6, 28.4) |
|  | aTB | -0.42  (-1.56, 1.30) | 18.22  (11.97, 28.27) | 22.26  (16.19, 33.00) | -0.5  (-2.0, 1.2) | 20.5  (14.7, 25.4) | 24.1  (20.3, 29.0) |
|  | aTB + sTB | 2.06  (0.44, 5.29) | 25.69  (17.05, 38.72) | 31.24  (23.15, 45.18) | 1.5  (0.4, 3.4) | 20.3  (14.6, 25.2) | 23.9  (20.1, 28.8) |
|  | nTB | -2.75  (-4.59, -1.11) | 19.74  (12.62, 31.20) | 30.40  (18.23, 46.42) | -2.6  (-4.8, -0.9) | 18.9  (13.5, 23.7) | 27.6  (24.8, 32.6) |
|  | TB deaths | 0.41  (0.28, 0.60) | 1.16  (0.75, 1.62) | 1.37  (1.03, 1.89) | 6.3  (5.1, 8.5) | 18.4  (13.0, 23.0) | 21.8  (17.9, 26.4) |
| **Zero infectiousness**  **(0)** | sTB | 5.31  (4.81, 6.45) | 6.61  (5.99, 7.79) | 8.35  (7.58, 9.71) | 13.3  (12.7, 14.4) | 16.5  (15.6, 17.6) | 20.8  (19.8, 21.7) |
|  | aTB | 7.23  (6.39, 9.75) | 17.11  (15.42, 21.85) | 21.66  (19.60, 27.10) | 7.1  (6.7, 8.0) | 16.8  (16.0, 17.9) | 21.2  (20.3, 22.2) |
|  | aTB + sTB | 12.51  (11.19, 16.21) | 23.71  (21.41, 29.66) | 30  (27.14, 36.84) | 8.8  (8.4, 9.7) | 16.7  (15.9, 17.8) | 21.1  (20.2, 22.1) |
|  | nTB | 10.88  (9.36, 14.78) | 21.24  (18.88, 27.21) | 36.3  (32.43, 45.55) | 7.6  (7.1, 8.6) | 14.9  (14.1, 16.0) | 25.5  (24.3, 26.6) |
|  | TB deaths | 0.76  (0.7, 0.95) | 0.95  (0.87, 1.16) | 1.19  (1.10, 1.44) | 12.2  (11.7, 13.3) | 15.2  (14.3, 16.2) | 19.1  (18.1, 20.0) |

*Abbreviations: nTB = non-infectious TB; aTB = infectious asymptomatic TB; sTB = infectious symptomatic TB*

## Sensitivity analysis results: vaccines effective with any or current infection status including efficacy in pre-disease stages

Figure O and Table K shows the short-term vs. longer-term vaccine impact where an any infection or current infection vaccine is also effective in early disease stages.

If the vaccine preventing progression to any infectious disease was also effective if delivered to those with nTB, and the vaccine preventing progression to infectious symptomatic disease only was also effective if delivered to those with nTB and aTB, we observed differences in impact compared to any infection vaccines that were not effective if given to individuals with nTB and aTB.

The impact of a vaccine preventing any infectious disease effective if given to individuals with nTB increased for all outcomes compared to the vaccine that was ineffective if given to individuals with nTB, in both the short- and longer-term, as a higher proportion of the population would receive protection from the vaccine (Figure O, Table K). In both the short- and longer-term, this increase resulted in the impact of this vaccine being as much as or greater than the impact of a vaccine preventing progression to any disease for all outcomes (Figure O, Table K).

The impact of a vaccine preventing only infectious symptomatic disease that was effective if given to individuals with nTB or aTB became more pronounced compared to the vaccine that was ineffective if given to individuals with nTB or aTB, with greater increases in nTB or aTB episodes, and greater reductions in sTB episodes and TB deaths (Figure O, Table K). In the short-term, the greatest impact on averting sTB episodes and TB deaths was from a vaccine preventing only infectious symptomatic disease effective if given to individuals with nTB or aTB, compared to a vaccine preventing any disease, or preventing any infectious disease effective if given to individuals with nTB. However, in the longer term, the relative impact of the vaccine decreases compared to the other vaccines, due to the build-up of the transmission effect (Figure O, Table K).

When comparing the impact for any infection vaccines effective if given to individuals with nTB or aTB, to current infection vaccines effective if given to individuals with nTB or aTB, we observed more pronounced differences between vaccines preventing any infectious disease and vaccines preventing any disease for current infection vaccines compared to any infection vaccines (Figure O, Table K).


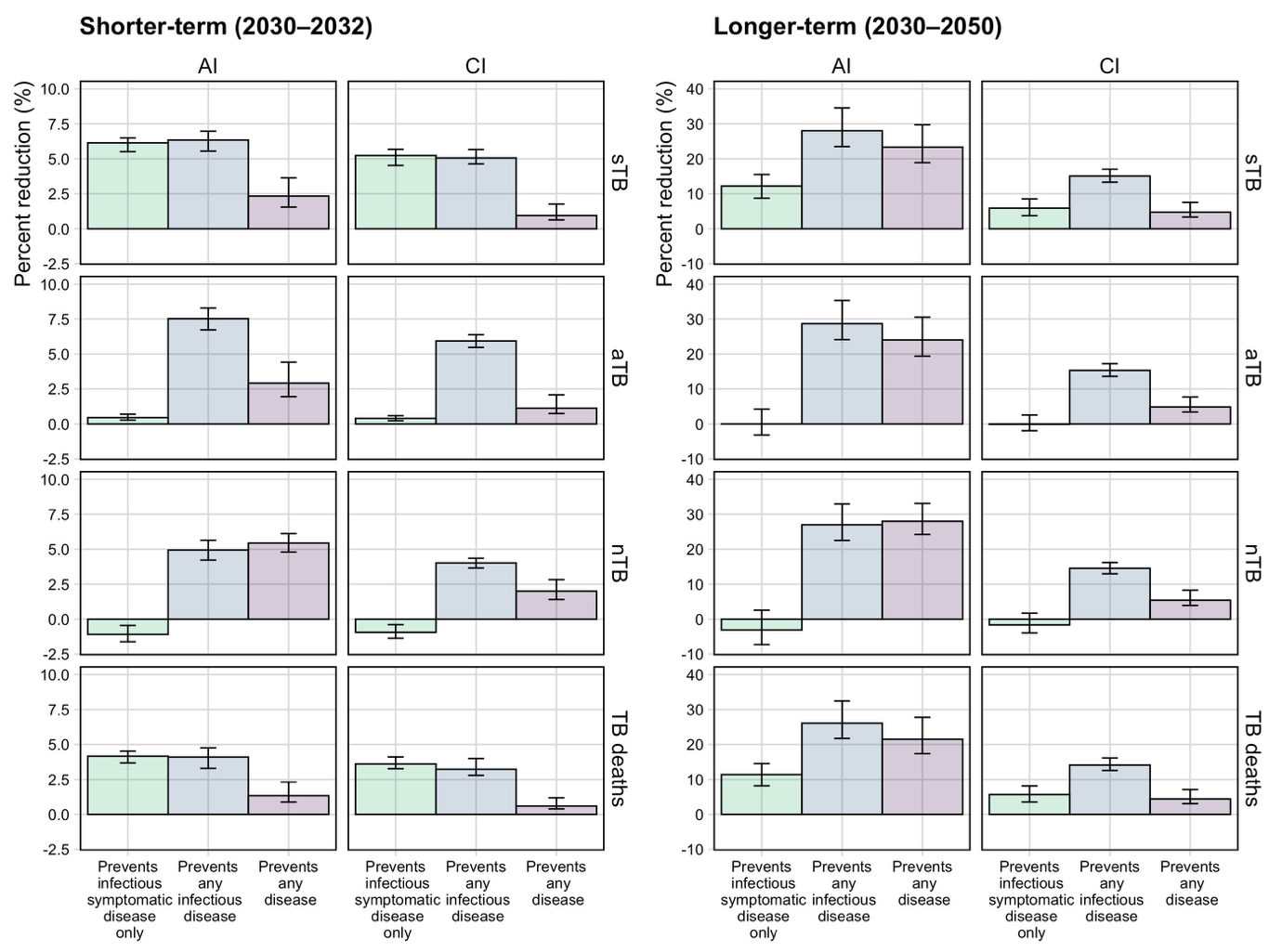


Figure O Percentage cumulative TB episodes and deaths averted between 2030–2032 (left column) or between 2030–2050 (right column), where vaccines were effective with any infection or current infection, including pre-symptomatic disease stages, with baseline (0.62, 1) infectiousness of aTB relative to sTB. Error bars represent 95% uncertainty intervals. Note y-axis scales differ. *Abbreviations: AI = any infection; aTB = infectious asymptomatic TB; CI = current infection; nTB = non-infectious TB; sTB = infectious symptomatic TB.*

Table K Number and percentage cumulative TB episodes and deaths averted (2030–2032 and 2030–2050) for vaccines effective in any infection or current infection, including disease stages, under baseline asymptomatic TB infectiousness relative to symptomatic TB.

|  |  | **AI (including disease)** | | | **CI (including disease)** | | |
| --- | --- | --- | --- | --- | --- | --- | --- |
| **Vaccine prevents progression to:** | | **Infectious symptomatic disease only** | **Any infectious disease** | **Any**  **disease** | **Infectious symptomatic disease only** | **Any infectious disease** | **Any**  **disease** |
| **Episodes Averted in millions (2030–2032)** | sTB | 0.40  (0.34, 0.49) | 0.42  (0.35, 0.53) | 0.15  (0.10, 0.27) | 0.34  (0.28, 0.41) | 0.33  (0.28, 0.41) | 0.06  (0.04, 0.13) |
|  | aTB | 0.07  (0.04, 0.13) | 1.25  (0.94, 1.54) | 0.47  (0.30, 0.78) | 0.06  (0.03, 0.11) | 0.97  (0.73, 1.20) | 0.18  (0.11, 0.37) |
|  | aTB + sTB | 0.47  (0.39, 0.61) | 1.66  (1.30, 2.07) | 0.62  (0.39, 1.06) | 0.41  (0.33, 0.51) | 1.30  (1.02, 1.60) | 0.24  (0.15, 0.50) |
|  | nTB | -0.21  (-0.27, -0.12) | 0.97  (0.63, 1.32) | 1.09  (0.66, 1.52) | -0.18  (-0.24, -0.10) | 0.79  (0.48, 1.11) | 0.40  (0.24, 0.59) |
|  | TB deaths | 0.05  (0.04, 0.06) | 0.04  (0.03, 0.06) | 0.01  (0.01, 0.03) | 0.04  (0.03, 0.05) | 0.04  (0.03, 0.05) | 0.01  (0, 0.01) |
| **% Reduction (2030–2032)** | sTB | 6.1  (5.5, 6.5) | 6.3  (5.5, 7.0) | 2.3  (1.5, 3.6) | 5.2  (4.5, 5.7) | 5.1  (4.6, 5.7) | 0.9  (0.6, 1.8) |
|  | aTB | 0.5  (0.3, 0.7) | 7.5  (6.7, 8.3) | 2.9  (1.9, 4.4) | 0.4  (0.2, 0.6) | 5.9  (5.5, 6.4) | 1.1  (0.7, 2.1) |
|  | aTB + sTB | 2.1  (2.0, 2.3) | 7.2  (6.4, 7.9) | 2.7  (1.8, 4.2) | 1.8  (1.7, 1.9) | 5.7  (5.2, 6.2) | 1.1  (0.7, 2.0) |
|  | nTB | -1.1  (-1.6, -0.4) | 4.9  (4.2, 5.6) | 5.4  (4.8, 6.1) | -0.9  (-1.4, -0.4) | 4.0  (3.7, 4.4) | 2.0  (1.4, 2.8) |
|  | TB deaths | 4.2  (3.7, 4.5) | 4.1  (3.3, 4.8) | 1.3  (0.9, 2.3) | 3.6  (3.3, 4.1) | 3.2  (2.8, 4.0) | 0.6  (0.4, 1.2) |
| **Episodes Averted in millions (2030–2050)** | sTB | 4.60  (2.97, 6.71) | 10.94  (8.27, 16.13) | 9.01  (6.69, 13.93) | 2.27  (1.26, 3.54) | 5.83  (4.46, 7.83) | 1.79  (1.20, 3.44) |
|  | aTB | 0.02  (-2.77, 4.84) | 27.95  (19.19, 39.43) | 23.21  (16.33, 33.63) | -0.09  (-1.65, 2.90) | 14.94  (10.07, 19.61) | 4.68  (3.09, 8.28) |
|  | aTB + sTB | 4.76  (0.8, 11.26) | 38.94  (27.56, 54.91) | 32.3  (22.89, 47.11) | 2.29  (-0.16, 6.42) | 20.79  (14.56, 27.48) | 6.49  (4.34, 11.76) |
|  | nTB | -3.80  (-7.48, 4.06) | 31.72  (18.2, 43.96) | 33.23  (18.71, 47.14) | -1.96  (-4.02, 2.71) | 17.24  (8.94, 24.55) | 6.64  (3.67, 10.18) |
|  | TB deaths | 0.73  (0.45, 1.02) | 1.69  (1.26, 2.37) | 1.38  (1.03, 2) | 0.36  (0.2, 0.55) | 0.91  (0.69, 1.22) | 0.27  (0.19, 0.51) |
| **% Reduction (2030–2050)** | sTB | 12.2  (8.7, 15.5) | 28.0  (23.5, 34.5) | 23.3  (18.9, 29.7) | 5.9  (3.7, 8.5) | 15.0  (13.3, 17.0) | 4.7  (3.3, 7.5) |
|  | aTB | 0.0  (-3.1, 4.2) | 28.7  (24.1, 35.4) | 24.0  (19.4, 30.5) | -0.1  (-1.9, 2.6) | 15.3  (13.6, 17.3) | 4.9  (3.4, 7.7) |
|  | aTB + sTB | 3.5  (0.6, 7.3) | 28.5  (23.9, 35.1) | 23.8  (19.2, 30.3) | 1.6  (-0.1, 4.1) | 15.2  (13.5, 17.2) | 4.8  (3.4, 7.6) |
|  | nTB | -3.1  (-7.3, 2.6) | 27.0  (22.5, 33.0) | 28.0  (24.2, 33.1) | -1.6  (-3.9, 1.7) | 14.6  (13.0, 16.2) | 5.4  (3.9, 8.3) |
|  | TB deaths | 11.4  (8.2, 14.5) | 26.1  (21.7, 32.5) | 21.5  (17.4, 27.7) | 5.7  (3.5, 8.1) | 14.1  (12.6, 16.1) | 4.4  (3.1, 7.1) |

*Abbreviations: nTB = non-infectious TB; aTB = infectious asymptomatic TB; sTB = infectious symptomatic TB*

## Sensitivity analysis results: scenarios with varying vaccine efficacy


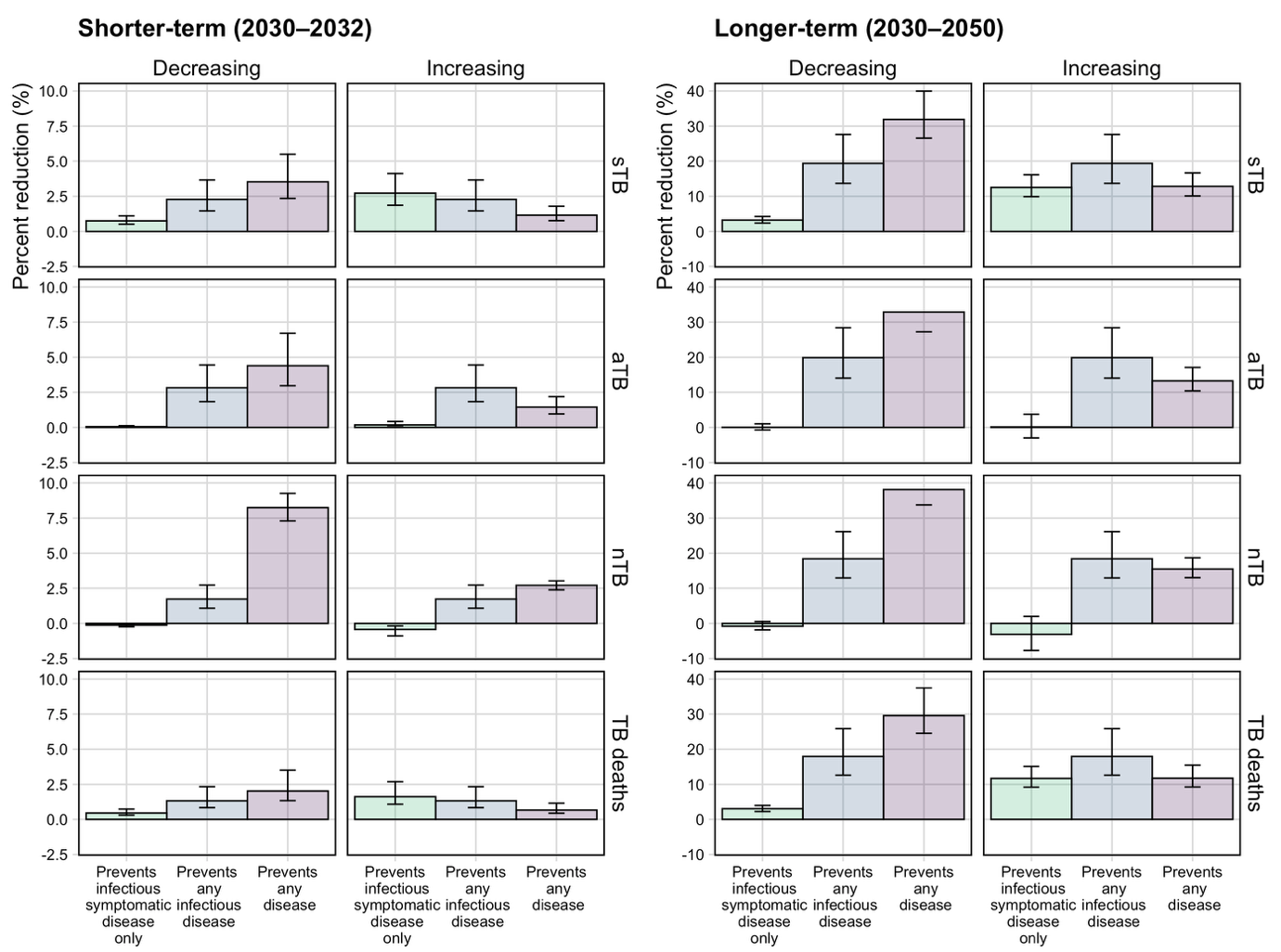


Figure P Percentage cumulative TB episodes and deaths averted between 2030–2032 (left column) or between 2030–2050 (right column). Error bars represent 95% uncertainty intervals. Note y-axis scales differ. *Abbreviations: aTB = infectious asymptomatic TB; nTB = non-infectious TB; sTB = infectious symptomatic TB.*

## Sensitivity analysis results: scenarios with varying duration of protection

**
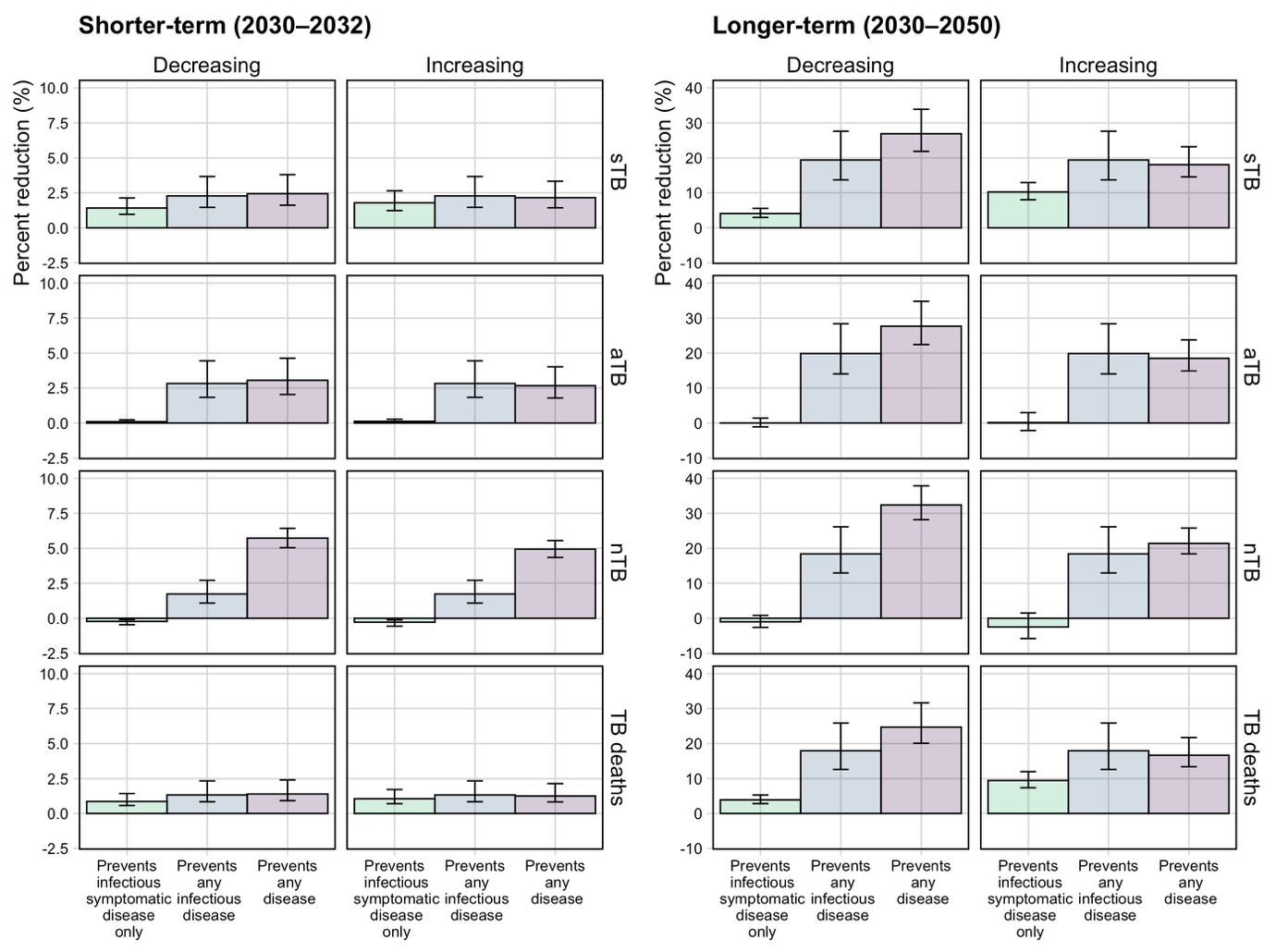
**

Figure Q Percentage cumulative TB episodes and deaths averted between 2030–2032 (left column) or between 2030–2050 (right column). Error bars represent 95% uncertainty intervals. Note y-axis scales differ. *Abbreviations: aTB = infectious asymptomatic TB; nTB = non-infectious TB; sTB = infectious symptomatic TB.*

**References**

1. Clark RA, Weerasuriya CK, Portnoy A, Mukandavire C, Quaife M, Bakker R, et al. New tuberculosis vaccines in India: modelling the potential health and economic impacts of adolescent/adult vaccination with M72/AS01E and BCG-revaccination. BMC Med. 2023 Aug 4;21(1):288.

2. Richards AS, Sossen B, Emery JC, Horton KC, Heinsohn T, Frascella B, et al. Quantifying progression and regression across the spectrum of pulmonary tuberculosis: a data synthesis study. Lancet Glob Health. 2023 May 1;11(5):e684–92.

3. Horton KC, Richards AS, Emery JC, Esmail H, Houben RMGJ. Reevaluating progression and pathways following Mycobacterium tuberculosis infection within the spectrum of tuberculosis. Proc Natl Acad Sci. 2023 Nov 21;120(47):e2221186120.

4. Asymptomatic TB [Internet]. [cited 2025 Feb 12]. Available from: https://www.who.int/teams/global-tuberculosis-programme/tb-reports/global-tuberculosis-report-2024/featured-topics/asymptomatic-tb

5. United Nations. Department of Economic and Social Affairs, Population Division. World Population Projections [2022 Revision] [Internet]. 2022 [cited 2025 Jan 23]. Available from: https://population.un.org/wpp/Download/Standard/Population/

6. Prem K, Zandvoort K van, Klepac P, Eggo RM, Davies NG, Group C for the MM of IDC 19 W, et al. Projecting contact matrices in 177 geographical regions: An update and comparison with empirical data for the COVID-19 era. PLOS Comput Biol. 2021 July 26;17(7):e1009098.

7. World Health Organization. WHO TB burden estimates [2022 revisions] [Internet]. 2022 [cited 2025 Jan 23]. Available from: https://www.who.int/tb/country/data/download/en/

8. World Health Organization. Case Notifications [Internet]. [cited 2025 Jan 23]. Available from: https://www.who.int/tb/country/data/download/en/

9. Emery JC, Dodd PJ, Banu S, Frascella B, Garden FL, Horton KC, et al. Estimating the contribution of subclinical tuberculosis disease to transmission: An individual patient data analysis from prevalence surveys. Kana BD, editor. eLife. 2023 Dec 18;12:e82469.

10. Andrews JR, Noubary F, Walensky RP, Cerda R, Losina E, Horsburgh CR. Risk of Progression to Active Tuberculosis Following Reinfection With Mycobacterium tuberculosis. Clin Infect Dis. 2012 Mar 15;54(6):784–91.

11. Glynn JR, Murray J, Bester A, Nelson G, Shearer S, Sonnenberg P. High Rates of Recurrence in HIV-Infected and HIV-Uninfected Patients with Tuberculosis. J Infect Dis. 2010 Mar 1;201(5):704–11.

12. Organization WH. Guidelines for treatment of drug-susceptible tuberculosis and patient care [Internet]. World Health Organization; 2017 [cited 2025 Jan 23]. Available from: https://iris.who.int/handle/10665/255052

13. Organization WH. Treatment of drug-susceptible tuberculosis: rapid communication [Internet]. World Health Organization; 2021 [cited 2025 Jan 23]. Available from: https://iris.who.int/handle/10665/341729

14. World Health Organization. Treatment Outcomes [Internet]. [cited 2025 Jan 23]. Available from: https://www.who.int/tb/country/data/download/en/

15. Rajagopalan S. Tuberculosis and aging: a global health problem. Clin Infect Dis Off Publ Infect Dis Soc Am. 2001 Oct 1;33(7):1034–9.

16. Clark RA, Mukandavire C, Portnoy A, Weerasuriya CK, Deol A, Scarponi D, et al. The impact of alternative delivery strategies for novel tuberculosis vaccines in low-income and middle-income countries: a modelling study. Lancet Glob Health. 2023 Apr 1;11(4):e546–55.

17. World Health Organization. Global Tuberculosis Report 2020. 2020.

18. Andrianakis I, Vernon IR, McCreesh N, McKinley TJ, Oakley JE, Nsubuga RN, et al. Bayesian History Matching of Complex Infectious Disease Models Using Emulation: A Tutorial and a Case Study on HIV in Uganda. PLOS Comput Biol. 2015 Jan 8;11(1):e1003968.

19. Goldstein M. Bayes Linear Analysis for Complex Physical Systems Modeled by Computer Simulators. In: Dienstfrey AM, Boisvert RF, editors. Uncertainty Quantification in Scientific Computing [Internet]. Berlin, Heidelberg: Springer Berlin Heidelberg; 2012 [cited 2022 Apr 5]. p. 78–94. (IFIP Advances in Information and Communication Technology; vol. 377). Available from: http://link.springer.com/10.1007/978-3-642-32677-6_6

20. Williamson D, Goldstein M, Allison L, Blaker A, Challenor P, Jackson L, et al. History matching for exploring and reducing climate model parameter space using observations and a large perturbed physics ensemble. Clim Dyn. 2013 Oct 1;41:1703–29.

21. Iskauskas A, McKinley TJ. hmer: History Matching and Emulation Package [Internet]. 2024 [cited 2025 Jan 15]. Available from: https://cran.r-project.org/web/packages/hmer/index.html

22. Indian Council of Medical Research. Central TB Division, Ministry of Health and Family Welfare, Government of India. National TB prevalence survey in India. [Internet]. [cited 2025 Jan 15]. Available from: https://tbcindia.mohfw.gov.in/wp-content/uploads/2023/05/25032022161020NATBPSReport.pdf

23. Pandey S, Chadha VK, Laxminarayan R, Arinaminpathy N. Estimating tuberculosis incidence from primary survey data: a mathematical modeling approach. Int J Tuberc Lung Dis. 2017 Apr;21(4):366–74.

24. World Health Organization. WHO TB incidence estimates disaggregated by age group, sex and risk factor [Internet]. [cited 2025 Jan 23]. Available from: https://www.who.int/tb/country/data/download/en/

25. Mandal S, Chadha VK, Laxminarayan R, Arinaminpathy N. Counting the lives saved by DOTS in India: a model-based approach. BMC Med. 2017 Mar 3;15(1):47.

26. Tait DR, Hatherill M, Van Der Meeren O, Ginsberg AM, Van Brakel E, Salaun B, et al. Final Analysis of a Trial of M72/AS01E Vaccine to Prevent Tuberculosis. N Engl J Med. 2019;381(25):2429–39.

27. HPV information centre. South Africa: Human Papillomavirus and Related Cancers, Fact Sheet 2016 (2016-12-15). [Internet]. 2016 [cited 2016 Dec 17]. Available from: https:// www.hpvcentre.net/statistics/reports/ZAF_FS.pdf

28. UNESCO Institute for Statistics. Education: gross enrolment ratio by level of education [Internet]. 2016 [cited 2017 Jan 3]. Available from: http://data.uis.unesco.org/?queryid=142.

29. M. Harouna Djingarey. Roll out of the meningococcal A conjugate vaccine through mass vaccination campaigns in countries of the African meningitis belt [Internet]. 2014 [cited 2016 Nov 28]. Available from: http://www.who.int/immunization/sage/meetings/2014/october/2.DJINGAREY_Session6_SAGE_Oct2014_FINAL_21Oct2014.pdf?ua=1
